# Access to Healthcare Services for the Deaf: A Scoping Review of Reviews

**DOI:** 10.1111/hex.70554

**Published:** 2026-01-26

**Authors:** Marie‐Mychèle Pratte, Magaly Brodeur, Marie‐Eve Perron, Catherine Hudon

**Affiliations:** ^1^ Department of Family Medicine and Emergency Medicine Université de Sherbrooke Sherbrooke Quebec Canada; ^2^ Ingram School of Nursing Faculty of Medicine and Health Sciences, McGill University Montreal Quebec Canada; ^3^ Mary's Research Center, Hayes Pavilion Montreal Quebec Canada

**Keywords:** access, deaf, health inequities, health services, healthcare services, patient‐oriented research, review

## Abstract

**Background:**

Cultural and linguistic minorities are at a higher risk of poorer health, poorer health outcomes, and poorer quality of care, in part due to a lack of access to healthcare services. Inequities in access to healthcare services have been found in several Deaf populations and are associated with poorer physical and mental health outcomes.

**Objective:**

This review aims to describe the breadth, scope, and nature of the literature on access to healthcare services for Deaf adults.

**Methods:**

A scoping review was conducted according to Arksey and O'Malley. Ten scientific databases and grey literature were searched for reviews published between 2000 and 2025 in English, French, American Sign Language, and Quebec Sign Language. A chart form was created for extraction. Results were analyzed using narrative synthesis and the Levesque et al. conceptual framework of access to healthcare. Research priorities were identified during a deliberative workshop with members of the Deaf community in Québec, Canada.

**Results:**

Eighteen reviews were included in this study. All dimensions of the conceptual framework have been explored (approachability, ability to perceive, acceptability, ability to seek, availability and accommodation, ability to reach, affordability, ability to pay, appropriateness, and ability to engage). Our analysis revealed new factors influencing access that need to be considered: cultural competence, communication barriers, sociodemographic characteristics, and technological support. We also identified that the social inclusion of the Deaf impacts multiple dimensions in a cross‐sectional manner.

**Conclusion:**

The Deaf face barriers to accessing healthcare services at every stage of their pathway. This review has identified several areas of access where further research is needed to address the disparities experienced by Deaf communities. There is an urgent need to involve the Deaf community in shaping the research on their access to healthcare services.

**Patient or Public Contribution:**

A Deaf patient partner contributed as a co‐researcher to the study design, the conduct of the review, and the interpretation of the data. This collaboration helped to contextualise and interpret the findings of the review.

## 1 Introduction

Cultural and linguistic minorities are at a higher risk of poorer health, poorer health outcomes, and poorer quality of care [1, 2, 3]. Lack of access to healthcare services has been identified as one of the main contributors to these disparities [1, 2, 3, 4]. Access to healthcare services (hereafter referred to as access) is “the opportunity to reach and obtain appropriate healthcare services in situations of perceived need for care.” [5] Inequalities in access can occur at any point in the utilisation pathway: identifying healthcare needs, seeking healthcare, reaching healthcare services, and receiving quality healthcare services that meet their healthcare needs [5].

Inequities in access have been identified in several Deaf populations [6, 7, 8]. Deaf with a capital ‘D’ describes people who primarily communicate with Sign Language, identify as part of the Deaf community, and engage in Deaf culture and society [9]. In contrast, deaf with a lowercase ‘d’ refers to individuals who are medically deaf but do not identify as part of the Deaf community [9]. The Deaf perceive that they receive a lower quality of care, which leads to frustration during interactions with healthcare professionals and the healthcare system [10, 11]. Over time, this frustration can lead to distrust and avoidance of healthcare, which can ultimately have a negative impact on their health [10, 11, 12, 13]. In addition, linguistic barriers experienced by Deaf patients have led to misdiagnosis [10, 12, 13].

Studies have shown that disparities in access are linked with worse health outcomes for the Deaf [2, 14, 15]. A recent systematic review highlighted poorer physical and mental health outcomes for the Deaf [2]. Many authors describe the Deaf community as underserved in health services and underrepresented in health research [10, 16, 17, 18]. The COVID‐19 pandemic has worsened access problems for Deaf patients [19, 20, 21]. Face mask making communication difficult, social distancing, and limited access to information and services have led to more isolation, poor understanding of prevention, and physical mental health concerns.

Despite increasing research efforts to understand the difficulties experienced by the Deaf and to improve their access, systemic barriers remain, with communication barriers being the most prominent [22, 23, 24]. These communication barriers extend beyond individual interactions; they permeate broader systemic, organisational, and societal levels, affecting how information is disseminated and how services are structured [25, 26, 27, 28, 29, 30, 31, 32, 33, 34, 35]. This review of reviews aims to describe the breadth, scope, and nature of the available literature on access to healthcare services for the Deaf. The objectives are to identify which aspects of access have been studied and the limitations of the available literature.

## 2 Methods

We conducted a scoping review using Arksey and O'Malley's methodological framework [36], enhanced by Levac et al. [37] This methodology allows for describing the extent and scope of published literature while including grey literature. It also allows researchers to adapt their methodology as they become familiar with the literature [36]. The framework involves six iterative steps: (1) identifying the research question, (2) identifying relevant studies, (3) selecting studies, (4) charting the data, (5) collating, summarising, and reporting the results, and (6) consulting with citizens. The scoping review methodology was adapted for a scoping review of reviews according to the recommendations of Schultz et al. [38] The Preferred Reporting Items of Systematic Reviews and Meta‐analysis extension for scoping reviews (PRISMA‐ScR) guided the reporting of this review (see Supporting Information S1: File 1) [39]. A protocol was published on OSF [40].

### 2.1 Patient Involvement in This Review

A Deaf patient partner was recruited to collaborate as a co‐researcher taking into account his experiential knowledge. His involvement shaped the research question and contextualised the findings. His level of involvement varied according to the needs of the project, his interests, and the budget. His involvement is detailed in the description of each step of the scoping review.

### 2.2 Conceptual Framework

**Figure 1:**
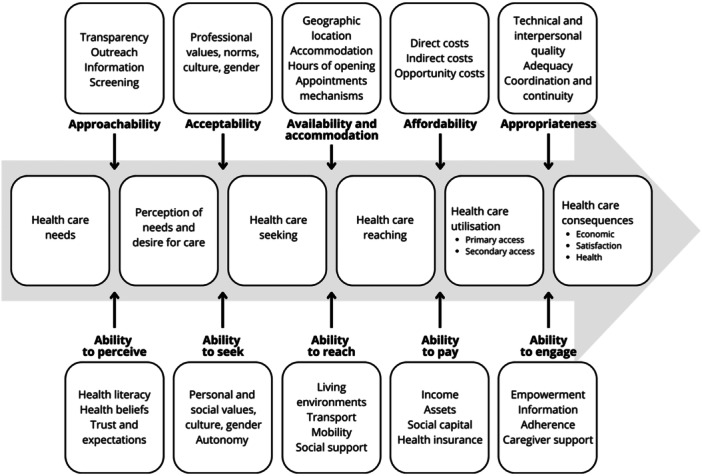
Conceptual framework of access to healthcare by Levesque et al. [5].

For this review, the conceptual framework of access to healthcare by Levesque et al. [5] was chosen (Figure 1). As described by the authors, access is a complex process that results from the interaction of supply side accessibility and demand side abilities [5]. On the supply side, the accessibility of health services is determined by the characteristics of the providers, organisations, and systems that deliver health services. Accessibility is divided into five dimensions: approachability, acceptability, availability and accommodation, affordability, and appropriateness. The dimensions on the demand side, which represent the abilities of individuals, communities, and populations, are the ability to perceive, the ability to seek, the ability to reach, the ability to pay, and the ability to engage [5].

### 2.3 Identifying the Research Question

The review question was: “What do reviews say about access to healthcare services for the Deaf?” The PICo criteria were used to further clarify the focus of the review: (1) Population: Deaf adults, (2) Phenomena of interest: access, and (3) Context: healthcare services [41].

### 2.4 Identifying Relevant Studies

We identified relevant studies using scientific databases, grey literature, and additional sources. A comprehensive search strategy was developed in collaboration with an academic health librarian and the patient partner. The search strategy was based on the PICo identified in Step 1. During the initial searches, we identified several discrepancies in the description of the population between the title or abstract and the full‐text. For example, an article would use “hard‐of‐hearing patients” in its title or abstract but then describe its population as “Deaf patients”. Therefore, our search strategy was adapted to include keywords related to the deaf, hard‐of‐hearing, and people with hearing loss or hearing impairment to ensure that all relevant reviews were retrieved by the search strategy even if this resulted in a significantly larger number of articles to review. The search string developed was then adapted for each database (see Supporting Information S2: File S2). MMP conducted the searches in 10 scientific databases (MEDLINE, CINAHL, Academic Search Complete, APA PsycInfo, AMED, Health care Administration Database, ABI/INFORM Collection, SCOPUS, Cochrane Database of Systematic Reviews, and Epistemonikos) on February 24, 2024, for published reviews. On April 4, 2025, MMP updated the search in the databases to identify possible new reviews.

The grey literature search was conducted by MMP and the patient partner from February to April 2024. It included a search for reports and other publications from Deaf advocacy and research groups, a search in Theses Canada and the Gallaudet University Dissertation Registry, and a search of Deaf community news sources. We each divided the searches according to our language skills: the patient partner focused on American and Quebec Sign Language sources, and MMP focused on English sources. French sources were searched for by both the patient partner and MMP. For additional sources, citation tracking and screening of reference lists of selected reviews were undertaken by MMP. A manual search of scientific journals specialising in the publication of articles related to the Deaf community was also conducted by MMP.

A search of Google Scholar was also carried out by MMP on April 29, 2024, with an adapted search string to see if any additional relevant reviews could be identified in the first 10 pages of the results (see Supporting Information S2: File S2). The Google Scholar search was carried out again on April 14, 2025, by MMP. Sources were imported into Covidence (Veritas Health Innovation, Melbourne, Australia) for citation management. Duplicates were automatically and manually removed after import.

### 2.5 Selecting the Studies

The research team (MMP, MB, CH, and the patient partner) met to discuss the selection process and the inclusion and exclusion criteria before starting the study selection. The inclusion criteria for this scoping review are presented in Table 1.

**Table 1:** Inclusion and exclusion criteria.

Criteria	Inclusion	Exclusion
Population	Deaf adults Subgroup of Deaf adults (e.g., Deaf women)	Deaf, hard‐of‐hearing or individuals with hearing impairments Under 18 years old Deaf populations and other groups (e.g., multiple linguistic minorities, people with disabilities)
Phenomena of interest	Access to health services and/or at least one dimension of access according to Levesque et al. [5]	
Context	Healthcare services provided to an individual, a group, a community, or a population.	Services or care offered outside the healthcare system (e.g., dental, orthodontic or optometry services) Health services given in the justice system (e.g., forensic assessments) Diagnosis or treatment of deafness
Type of documents	Reviews	Primary articles, clinical guidelines, protocols, and historical reviews
Other	From January 1, 2000, to April 4, 2025	Full‐text not available in English, French, ASL or LSQ

**Figure 2:**
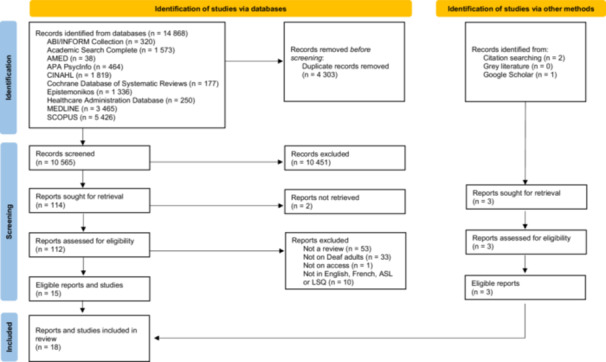
PRISMA flow diagram of study selection.

Study selection in Covidence followed a two‐stage process: title and abstract review and full‐text review (see Figure 2 for PRISMA flow diagram [42]). A pilot round of the title and abstract review of 20 randomly selected articles was conducted independently by MMP and MEP. The two reviewers met to compare the results and resolve disagreements through consensus‐based discussion. The inclusion criteria were checked for minor clarifications after validation by CH and MB. The remaining articles were then screened by MMP. Articles lacking abstracts were automatically selected for full‐text reviews. The full‐text review was carried out independently by MMP and MEP. Any disagreements were resolved by a consensus‐based discussion with CH and MB. MMP attempted to contact the authors of the review to obtain clarification when the population studied was unclear. After two unsuccessful attempts to contact the authors, the articles were excluded.

### 2.6 Charting the Data

A Covidence extraction form was developed by MMP to extract data from the reviews (see Supporting Information S3: File S3). The extraction guidelines were detailed in the Covidence extraction form. If the information could not be identified, it was coded as “N/A” for “not available”. A pilot process of the extraction form was conducted by MMP for three randomly selected reviews. Following the pilot, MMP completed the extraction, which was validated by MEP.

### 2.7 Collating, Summarising, and Reporting the Results

A narrative synthesis of the data was carried out following the methodological framework of Popay et al. [43] This method allows the articles to be grouped even if their results are very heterogeneous, which facilitates the production of the synthesis and the exploration of relationships between the reviews and possible gaps. A narrative synthesis involves four steps: identifying a conceptual model (Levesque et al. [5] framework), developing a preliminary descriptive synthesis, exploring relationships in the data, and assessing the robustness of the synthesis [43]. For the preliminary descriptive synthesis, a textual description of each study was developed. This provided further familiarity with the reviews and helped to outline similarities or differences. The reviews were grouped according to methodology and type of health service. A table of descriptive characteristics was then created. Finally, a thematic translation was completed. A line‐by‐line coding of the “Results” section of each article was then carried out deductively from the dimensions and factors of the access conceptual framework [5]. An inductive approach was also used to identify new dimensions or factors. If some parts of the findings did not relate to the population or phenomena of interest, they were not included in the analysis. The descriptive characteristics of the reviews are presented using frequency tables. The components of access to healthcare services for the Deaf that were examined were identified based on Levesque et al.'s [5] conceptualisation of access to healthcare. The exploration of relationships was done through the data from individual articles or documents included in the selected reviews. To assess the robustness of the narrative synthesis, critical reflections and limitations of the process are presented in the discussion.

MMP conducted the synthesis under the supervision of CH and MB. Before starting the synthesis, MMP, MEP and the patient partner discussed their initial impression of the selected reviews, and which components of the narrative synthesis were more suitable for the analysis. In the middle and at the end of the synthesis, MMP and the patient partner met to discuss the synthesis and preliminary findings. This helped to gather insights, inform, and adjust the synthesis process.

### 2.8 Consulting with Deaf Citizens

The consultation aimed to complement the findings of the synthesis. A deliberative workshop approach was chosen because it allows for both informed and in‐depth discussions [44]. Eight Deaf participants (including the patient partner) were recruited using convenience sampling [45]. Recruitment was done through an Quebec Sign Language video and in a poster format in French, which was shared with Deaf organisations and on the research team's social media.

Inclusion criteria for the workshop were individuals who self‐identified as Deaf, over 18 years of age, and who had used healthcare services at least once in the past 2 years. Participants received information about the workshop and consent forms in advance in Quebec Sign Language and in written French. The workshop took place in three phases during a single activity lasting about 3 h: (1) presentation of results; (2) exchange between participants to develop their understanding; and (3) group deliberation: ranking research priorities according to their level of importance. The workshop was co‐facilitated by the patient partner and MMP. The data collected from the workshop were sociodemographic questionnaires and research priorities as well as their rankings. All the collected data was anonymized and denominated from the moment it was collected. There were no audio or visual recordings made of the workshop. Participants could withdraw from the activity at any moment and still receive the full compensation offered, a 75$ Visa gift card.

## 3 Results

From the 10 databases, 14,868 records were identified. After removing 4303 duplicates, 10,565 documents were screened. During the title and abstract review, 10,451 documents were excluded, which left 114 documents for full‐text review. Of these, two full‐texts couldn't be retrieved; therefore, 112 full‐texts were screened for their eligibility and 97 were excluded. After the screening process, 15 reviews remained. Two additional reviews were found in the reference lists of the selected reviews and one from Google Scholar, for a total of 18 studies. The screening process is shown in Figure 2.

### 3.1 Characteristics of the Reviews

The 18 included reviews were published in English between 2010 and 2025 (see Table 2 for a descriptive summary). The reviews were published in the United States (*n* = 4) [46, 47, 48, 49], the United Kingdom (*n* = 4) [28, 35, 50, 51], Brazil (*n* = 3) [52, 53, 54], South Africa (*n* = 2) [55, 56], Switzerland (*n* = 2) [26, 34], Botswana (*n* = 1) [25], Malaysia (*n* = 1) [57], and New Zealand (*n* = 1) [58]. The methodology used was described in 16 of the reviews: systematic review (*n* = 6) [35, 49, 52, 53, 55, 56], scoping review (*n* = 4) [34, 46, 57], integrative review (*n* = 3) [25, 28, 54], critical review (*n* = 1) [47], meta‐analysis (*n* = 1) [48], and qualitative synthesis (*n* = 1) [58].

**Table 2:** Descriptive summary of reviews.

First author, year Country	Aim	Type of review Number of studies	Definition of population	Main results (in relation to access)	Conclusions and recommendations (in relation to access)
Adigun et al., 2021 [55] South Africa	Identify and analyze the research on Deaf pregnant women and the antenatal care they receive	Systematic review *n* = 6	Women from the cultural‐linguistic Deaf community	∘ Communication challenges, limited access to Sign Language interpreters ∘ Accessibility, communication issues and geographical location of services impacted level of satisfaction ∘ Lower frequency of antenatal visits reported compared to hearing women	∘ Limited access to Sign Language interpreters could increase the risk of miscommunication with HCP and limit the understanding of Deaf women during their antenatal visits ∘ Infrequent antenatal care and risks of miscommunication increased the risks of pregnancy and/or delivery complications for Deaf pregnant women ∘ More research is needed to explore and investigate the current issues in antenatal care with a large sample size of Deaf pregnant women as active participants
Almeida et al., 2024 [52] Brazil	Identify the main obstacles for primary care HCP when caring for Deaf patients in Brazil	Systematic review *n* = 4	Deaf community members	∘ Lack of visual accessibility (signage, waiting rooms) ∘ Communication barriers due to a lack of professionals trained in Sign Language ∘ Communication barriers are also related to demographic and socioeconomic conditions ∘ Availability of services varies according to urban contexts	∘ The paucity of the literature seems to represent a lack of interest from the academic community and a lack of discussion in the scientific community and in civil society ∘ There is a need to prepare new HCP with technical capacity in Sign Language ∘ Proposals with the aim of providing full accessibility for the Deaf are needed
Baratedi et al., 2022 [25] Botswana	Identify the experiences of the Deaf accessing healthcare services in sub‐Saharan Africa and identify the barriers and facilitators when accessing healthcare services	Integrative review *n* = 7	Individuals who communicate through Sign Language and are culturally Deaf	∘ Ineffective communication is the major obstacle when accessing services ∘ Communication barriers are exacerbated by a lack of Sign Language interpreter services ∘ Lower education level and socio‐economic situation impacted access ∘ Deaf patients experienced discrimination and negative attitudes from HCP ∘ Non‐involvement in their care and privacy concerns were noted	∘ Deaf awareness, Sign Language training, and courses on ethics and human rights are needed for HCP ∘ Vernacular languages must also be considered and translated into Sign Language ∘ Interpreters should be present in health facilities to assist Deaf patients at every point of care ∘ Governemental waivers for out‐of‐pocket fees should be considered for the Deaf
Barbosa et al., 2025 [53] Brazil	Identify knowledge and attitudes of Deaf women in relation to contraceptive methods	Systematic review *n* = 12	Individuals who communicate through Sign Language and are culturally Deaf	∘ Deaf women had less knowledge on effective contraceptive methods ∘ Barriers to the use of contraceptive: communication barriers (including, lack of access to Sign Language interpreters), financial barriers, lack of access to sexual and reproductive health services, health beliefs, level of autonomy from family members and family attitudes	∘ Identified a need for research of higher level of evidence and interventional studies ∘ Reducing communication barriers would improve access and informed decision‐making
Flower et al., 2024 [50] United Kingdom	Identify the barriers older Deaf people face when accessing healthcare and explore the issues surrounding dementia for older Deaf people	Narrative literature review *n* = 26 (and 8 in‐depth key informant interviews)	Deaf individuals who identify themselves as a linguistic minority	∘ Care for health issues may be delayed or avoided because of previous experiences ∘ Access challenges: appointment mechanism, visual accommodations, lack of health information in Sign Language, lack of Deaf awareness by HCP, lack of Sign Language interpreters during appointments and in care homes ∘ Inappropriate dementia assessment, few specialist memory clinics, lack of care homes with staff with Sign Language competency ∘ Deaf older adults may experience more isolation from progressing dementia because of communication issues and being rejected by their community ∘ Deaf caregivers have limited access to information in Sign Language and accessible support group	∘ The prevalence of dementia in older Deaf is difficult to estimate due to a lack of accurate statistics ∘ Research should specify whether their participants self‐identify as culturally Deaf or not ∘ Cultural insiders could insure a more adequate representation of the diversity of Deaf populations in future research ∘ Deaf awareness training is needed for health and social care staff ∘ Screening and assessments for dementia must be linguistically appropriate, culturally sensitive and tailored to Deaf individuals ∘ More Deaf representation in healthcare and increased involvement in research could improve awareness and health outcomes for the Deaf
Flynn, 2020 [46] United States	Identify the use of the term “trust” (and its derivatives) around Deaf patients and their HCP	Scoping review *n* = 51	Individuals who identify as culturally Deaf	∘ Trust was rarely used in the literature in relation to the Deaf ∘ 11 of the 51 articles had significant discussions of trust. 22 articles had brief discussions of trust ∘ 51 articles discussed a trust‐related concept. The most prevalent were: communication, cultural competence, comfort and understanding	∘ A majority of the studies involved mental health services. Future research should be conducted in diverse settings to identify if the perspective of trust is influenced by the setting ∘ A large number of excluded studies focussed on cochlear implants. The author noted that this seemed to indicate that more emphasis in research seemed to be placed on pathologizing deafness rather than understanding the Deaf
Gould & Clark‐Howard, 2025 [58] New Zealand	Explore the experience of mental health therapies from the perspectives of Deaf clients, mental health practitioners and Sign Language interpreters	Qualitative synthesis *n* = 11	Individuals who identify as culturally Deaf	∘ Deaf clients: difficulties with Sign Language interpreters access, financial constraints, lack of mental health literacy, wanting their unicity respected within the community, facing audism, lack of trust in confidentiality processes ∘ Mental health practionners: lack of Deaf awareness, difficulties in adapting their therapeutic approaches, misdiagnosis and medication issues ∘ Sign Language interpreters: experiencing vicarious trauma, lack of emotional support because of confidentiality engagement	∘ There is a need for further research into the cultural and social considerations within mental health therapies for the Deaf ∘ Systemic solutions are needed to improve access for the Deaf ∘ Mental health practionners: Deaf awareness training and engagement with the Deaf community ∘ Sign Language interpreters: mental health training, clinical supervision and peer support are needed
Hanass‐Hancock & Satande, 2010 [56] South Africa	Investigate current knowledge and gaps regarding the Deaf and possible vulnerabilities to HIV infection, level of HIV/AIDS knowledge and access to HIV prevention and services	Systematic review *n* = 17	Deaf individuals	∘ The Deaf might have less HIV/AIDS knowledge, and have less access to HIV prevention and treatment services ∘ Lower utilisation of prevention services in some studies was explained due to communication difficulties, lack of accessible health information, confidentiality issues, and quality of care ∘ Community organisation can increase prevention services uptake	∘ Few studies have been conducted in countries with widespread HIV epidemics. ∘ Authors noted studies of varying methodological quality and an important need to improve ethical standards (e.g., independent Sign Language interpreters)
Hill et al., 2020 [47] United States	Identify potential barriers to care and provide some considerations for a future care model to improve care and delivery for Deaf patients	Critical review *n* = 34	Individuals who communicate through Sign Language and are culturally Deaf	∘ Barriers: poor cancer‐specific and preventive health literacy, lower levels of knowledge of cancer screening tests, lack of accessible and tailored healthcare resources for cancer‐specific information, and poor linguistic and cultural competency amongst HCP ∘ Linguistically and culturally adapted programmes strongly demonstrated improved cancer‐specific knowledge	∘ Deaf patients may present with more advanced stages of cancer ∘ Despite many studies presenting Deaf patients' frustration with the linguistic and cultural competency of HCP, only one study addressed this issue with medical students ∘ Guidelines for effective communication accessibility that consider the diverse cultural and linguistic needs of Deaf patients are needed ∘ There is a lack of literature on: treatment outcomes, disparities in rates, and adherence cancer‐specific guidelines
Kuenburg et al., 2016 [26] Switzerland	Summarise global aspects of the Deaf′s access to healthcare	N/A *n* = 98	Members of signing Deaf communities	∘ Challenges to access: communication barriers and lack of accessible health information ∘ The Deaf are at risk of further marginalisation because of intersecting identities (e.g., women, ethnic minorities, older adults, people with multiple and intellectual disabilities)	∘ Health needs of Deaf populations remain unmet globally ∘ Available literature mainly focuses on high‐income countries, Deaf populations in low and middle income countries might experience greater disparities ∘ Future HCP need to gain awareness of the needs of the Deaf and become competent in meeting those needs
Luton et al., 2022 [28] United Kingdom	Investigate the experiences of Deaf women in maternity and primary healthcare settings and identify barriers to access	Integrative review *n* = 11	Women who are Deaf community members	∘ Barriers: Lack of awareness of their rights when accessing healthcare, communication barriers, use of medical terminology by HCP, lack of Deaf awareness, and inaccessible processes (phone to appointments, no visual accessibility in facilities) ∘ Cultural and communication barriers may lead Deaf women to avoid or delay care	∘ HCP who engage with the Deaf community could help disseminate health education through peer interaction ∘ Deaf awareness training is recommended for HCP ∘ More research on the experiences of Deaf women in maternity care is needed
Mitchell, 2014 [48] United States	Investigate the magnitude of the knowledge‐related effect of interventions communicated in American Sign Language (ASL) in the Deaf population	Meta‐analysis *n* = 8	Deaf individuals who communicate primarily through ASL	∘ A significant knowledge related effect was found when health interventions are communicated in ASL (summary relative risk = 1.31)	∘ Similar health interventions should be implemented in large Deaf populations ∘ Further research needs to investigate the knowledge‐related effect of interventions communicated in other Signed Languages
Rivas Velarde et al., 2022 [34] Switzerland	Determine the state of available evidence regarding the use of VRI to overcome interpretation barriers and improve communication outcomes between Deaf patients and healthcare personnel	Scoping review *n* = 15	Deaf or hard of hearing Sign Language users	∘ Current literature isn′t sufficient to understand the use of VRI or its impact ∘ Available literature only comes from high‐income countries, although most Deaf populations live in low to middle income countries ∘ Advantages of VRI: help reach qualified Sign Language interpreters, sometimes preferred in non‐critical settings, better than lip‐reading and writing, removes delays from transportation, respected COVID‐19 in‐person restrictions ∘ Limitations of VRI: limited availability, not user‐friendly, technical problems, accessibility issues, largely unsatisfactory patient experiences	∘ Evidence of the efficiency of the use of VRI is insufficient ∘ VRI has the potential to address communication barriers if it is available along with other services and tools ∘ Technologies need to be developed centred around the views, needs, and rights of the Deaf community
Rogers et al., 2023 [35] United Kingdom	Investigate the efficacy and effectiveness of telemedicine interventions in comparison to face‐to‐face intervention for Deaf populations	Systematic review *n* = 2	Deaf individuals who communicate with Sign Language	∘ Available literature on this topic is limited and does not report completely on the effectiveness of the intervention. ∘ The 2 articles studied telemental health in the USA	∘ Telemedicine could help meet the Deaf′s cultural and linguistic needs considering that Deaf populations are dispersed geographically and the lack of language concordant therapists ∘ Higher quality research is needed face‐to‐face therapy comparator groups, including research on the satisfaction of patients and the acceptability of telemedicine for Deaf populations
Rogers et al., 2025 [51] United Kingdom	Identify the current knowledge on the perspectives of the Deaf and their experiences of healthcare	Scoping review *n* = 51	Deaf adult patients who are Sign Language users	∘ HCP do not meet the communication needs of their Deaf patients and lack cultural competence ∘ A lack of cultural competence can also create organisational and structural barriers to access ∘ On‐site interpreters promote patient‐HCP engagement and shared decision making	∘ Between Deaf populations, the inequalities in access to healthcare is a commonality ∘ Validated measure in Sign Language of the experience of Deaf people in healthcare are needed ∘ HCP need to ensure they provide high‐quality services while meeting the language, communication, and interaction needs of the Deaf
Souza et al., 2017 [54] Brazil	Analyze the main obstacles of healthcare access faced by the Deaf	Integrative review *n* = 24	Deaf community members who identify as part of a linguistic and cultural minority	∘ Communication barriers are present without a language broker, which impacts the creation of the alliance between patients and HCP ∘ Lower knowledge about self‐care, preventive guidance and poor access to health information ∘ Precarious public policies for the Deaf community	∘ Main obstacles in access to healthcare are related to linguistic barriers, due to a lack of training of HCP, financial difficulties when accessing Sign Language interpreters, and absence of accommodations ∘ Public health campaigns need to be planned more inclusively
Trout, 2018 [49] United States	Identify what information is available regarding the knowledge, opinions and perceptions of HIV/AIDS of members of the Deaf community	Systematic review *n* = 15	Deaf community members	∘ Deaf individuals had less knowledge about HIV/AIDS than the rest of the population regardless of the country of the study ∘ Communication barriers are present with HCP ∘ Lack of accessible health information about HIV/AIDS	∘ Future studies need to include Deaf individuals, Deaf medical workers, and Deaf researchers ∘ Studies on ways to create better communication between hearing HCP and their Deaf patients are needed ∘ More Deaf individuals working in the healthcare industry are needed
Yet et al., 2022 [57] Malaysia	Identify the communication methods used between Deaf patients and HCP	Scoping review *n* = 10	Individuals who identify as culturally Deaf and who communicate primarily through Sign Language	∘ Communication methods preferred by the Deaf varied ∘ Regardless of preference, writing and lip‐reading were often used ∘ The communication methods used were influenced by: experiences of Deaf patients, HCP cultural competence, and extrinsic factors (e.g., level of education, written language proficiency, and access to Sign Language interpreters)	∘ Multiple communication inadequacies between HCP and Deaf patients have been noted ∘ HCP need to increase their Deaf awareness and adopt a patient‐centred approach to prevent inadvertent harm ∘ Further research is needed to identify the gaps in Deaf patient‐HCP communication and to measure the effectiveness of each communication method on Deaf patients' understanding and health outcomes

Abbreviations: HCP, healthcare professionals; N/A, not available; VRI, video remote interpretation.

Each review included between 2 and 98 primary documents published between 1993 and 2024 for a total of 402 primary documents (median of 14 per review). Six of the reviews did not specify the country of publication of their sources (241 primary documents, 60% of primary documents) [26, 46, 47, 48, 52, 54] (Table 3). Of the remaining 161 primary documents, 90 are from the Americas, 39 from Africa, 20 from Europe, nine from Oceania, and three from Asia.

**Table 3:** Country of publication of primary documents included in the reviews.

Country	Frequency (%)
America	**90 (56)**
United States	77 (48)
Brazil	11 (6.8)
Canada	2 (1.2)
Africa	**39 (24.2)**
South Africa	13 (8.1)
Nigeria	9 (5.6)
Kenya	3 (1.9)
Ethiopia	3 (1.9)
Cameroon	2 (1.2)
Eswatini	2 (1.2)
Ghana	4 (2.5)
Uganda	1 (0.6)
Zimbabwe	1 (0.6)
Senegal	1 (0.6)
Europe	**20 (12.3)**
United Kingdom	12 (7.5)
Denmark	1 (0.6)
Ireland	1 (0.6)
Norway	1 (0.6)
Turkey	1 (0.6)
Germany	1 (0.6)
Italy	1 (0.6)
Spain	2 (1.2)
Oceania	**9 (5.6)**
Australia	6 (3.7)
New Zealand	3 (1.9)
Asia	**3 (1.8)**
Saudi Arabia	1 (0.6)
China	1 (0.6)
Malaysia	1 (0.6)

*Note:* The percentages are calculated on the 161 primary documents for which the country of origin was available. The values in bold represent the total for each continent.

### 3.2 Dimensions of Access to Healthcare Services Studied

**Figure 3:**
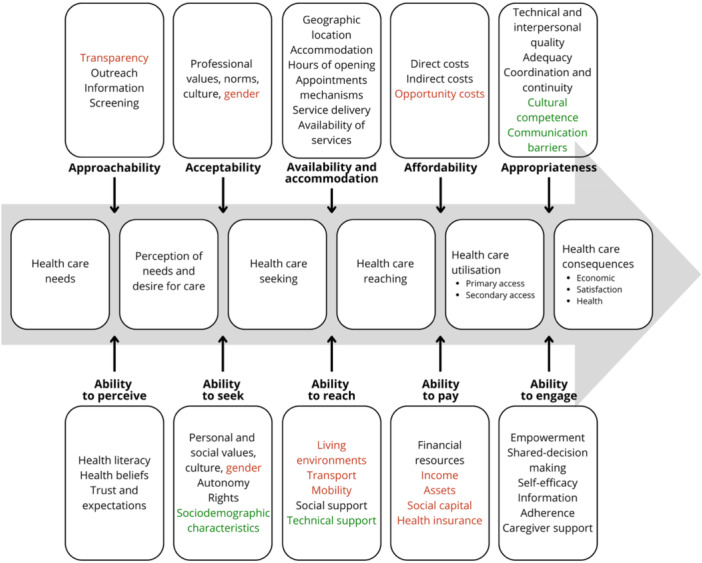
Factors influencing access studied. In black influencing factors studied, in red influencing factors that have not been studied, and in green newly identified influencing factors. Adapted from the conceptual framework of access to healthcare by Levesque et al. [5].

All the dimensions identified by Levesque et al. [5] were examined. Three reviews focused on one dimension of access [35, 46, 48]. The other 15 reviews studied between 3 and 10 dimensions, with only the review by Kuenburg et al. [26] covering all 10 dimensions [25, 26, 28, 34, 47, 49, 50, 51, 52, 53, 54, 55, 56, 57, 58]. The most studied dimension was appropriateness (*n* = 15) [25, 26, 28, 34, 47, 49, 50, 51, 52, 53, 54, 55, 56, 57, 58], and the least studied dimension was the ability to pay (*n* = 4) [25, 26, 34, 55]. Table 4 shows the full list of dimensions examined by each review. Figure 3 shows, for each of the 10 dimensions, which influencing factors were studied.

**Table 4:** Dimensions of access studied.

	Supply side					Demand side				
Authors, year	Approachability	Acceptability	Availability and accommodation	Affordability	Appropriateness	Ability to perceive	Ability to seek	Ability to reach	Ability to pay	Ability to engage
Adigun et al., 2021 [55]			X	X	X	X			X	X
Almeida et al., 2024 [52]	X		X	X	X		X			
Baratedi et al., 2022 [25]			X	X	X	X	X	X	X	X
Barbosa et al., 2025 [53]	X	X	X	X	X	X	X			X
Flower et al., 2024 [50]	X		X	X	X	X	X	X		X
Flynn, 2020 [46]						X				
Gould & Clark‐Howard, 2025 [58]	X	X	X	X	X	X	X	X		X
Hanass‐Hancock et Satande, 2010^57^	X	X			X	X		X		
Hill et all., 2020 [47]	X				X	X				X
Kuenburg et al., 2016 [26]	X	X	X	X	X	X	X	X	X	X
Luton et al., 2022 [28]	X	X	X		X	X	X			X
Mitchell, 2014 [48]	X									
Rivas Velarde et al., 2022 [34]					X			X	X	
Rogers et al., 2023 [35]			X							
Rogers et al., 2025 [51]	X		X		X			X		X
Souza et al., 2017 [54]	X				X	X				
Trout, 2018 [49]	X				X	X	X			X
Yet et al., 2022 [57]	X		X		X	X				X
*Frequency*	13	5	11	7	15	13	8	7	4	11

Communication barriers were found to affect most dimensions of access to healthcare services. Each dimension will be discussed separately in the following pages to highlight their specific impacts and contexts.

#### 3.2.1 Approachability

The availability of health information improves the approachability of health services. Deaf populations face a paucity of linguistically and culturally appropriate health information [26, 28, 47, 49, 50, 51, 52, 54, 56, 57, 58]. Because of this inaccessible health information, the Deaf may use different sources of information and tend to favour sources close to them, like their family, their friends and members of their Deaf community [26, 47, 49, 51, 53]. This would be important to consider when planning health promotion initiatives [50]. Inaccessible information about health services was also reported [26, 28, 47, 49, 50, 51, 53]. This shortage leads to lower access to preventative care information as well as lower knowledge about the healthcare services [26, 47, 53]. The lack of accessible information in Signed Languages has consequences for outreach services as well [51]. Some outreach initiatives and their importance have been reported [26, 50]. In terms of screening practices, there were differences in knowledge of screening services, leading to lower uptake of these services [47, 51, 56]. We did not identify information on the transparency of services.

#### 3.2.2 Ability to Perceive

Health beliefs and health literacy influence the perception of a need for care [5]. Lower levels of health literacy regarding general health topics, sexual and reproductive health, and cancer have been reported in several Deaf populations [25, 26, 28, 47, 49, 50, 53, 54, 56, 57, 58]. Accessible, culturally and linguistically appropriate interventions have been successful in improving health literacy [26, 30, 47, 49]. Health beliefs have been studied in relation to Human Immunodeficiency Virus, Acquired Immunodeficiency Syndrome, and dementia [26, 49, 50, 56]. After identifying a need for care, a desire for care is needed. Because of previous negative experiences with healthcare services, the Deaf frequently lack trust in healthcare professionals and anticipate receiving inferior care compared to the hearing majority [25, 26, 28, 46, 49, 50, 55, 57].

#### 3.2.3 Acceptability

The values, norms and culture of hearing healthcare professionals influence the acceptability of the services they provide and delay healthcare seeking [26, 28, 53, 56, 58]. Concerns regarding confidentiality and privacy have been raised, particularly when treatment occurs in open spaces [25, 53, 56, 58]. No information was found on how gender impacts the acceptability of services for the Deaf.

#### 3.2.4 Ability to Seek

Researchers have observed that some Deaf patients are unaware of their rights regarding access to healthcare services and the availability of interpreters [26, 28, 50]. Specific sociodemographic characteristics have also been found to affect the ability to seek healthcare services, notably socio‐economic status, educational level, and intersecting minority identities [26, 49, 52, 53, 58]. Some Deaf individuals have reported reliance on family members and family attitude as barriers to accessing care [53]. Religion or faith may also hinder access to sexual and reproductive healthcare services [53]. No information was found on how gender influences the ability to seek care.

#### 3.2.5 Availability and Accommodation

Distance from healthcare facilities, regional disparities, and a shortage of Deaf health professionals or those proficient in Sign Language remain significant barriers to accessing healthcare services [25, 26, 50, 52, 53, 55, 57, 58]. Specialised primary care centres for the Deaf have been implemented in Austria and France [26]. In the United Kingdom, a cognitive clinic specifically for British Sign Language users has been set up [50]. These dedicated centres provide full access and trained and employ skilled, knowledgeable staff [26, 50].

Telephone appointments continue to be offered to Deaf patients, even though they are unsuitable [50, 51]. Deaf individuals frequently have to attend appointments in person with an interpreter to receive test results [50, 51]. Telemedicine has the potential to reduce travel barriers by connecting patients with health professionals who are proficient in Sign Language, especially within mental health services [26, 35, 57]. Appointment systems and facilities often lack adequate accessibility, such as being limited to telephone bookings or not providing visual cues for calling the next patient [25, 26, 28, 50, 51, 52, 53]. Healthcare professionals who fail to consider the ways in which their organisation and services exclude Deaf patients display insufficient cultural competence and Deaf awareness [51]. There is also a lack of structural accommodation and reluctance among health personnel to use relay interpreting services [51]. Group support services for patients or caregivers are frequently inaccessible due to the absence of interpreters [50, 51].

#### 3.2.6 Ability to Reach

Researchers have found that technology, such as texting and relay Sign Language interpreting, can assist Deaf patients reaching health services [26, 34, 51]. Local organisations may promote the uptake of preventive and screening services [56, 58]. In long‐term care, family and friends often act as advocates to ensure the well‐being and needs of the Deaf residents [50]. There is no information available regarding the impact of housing, transport, or mobility on their ability to reach healthcare services.

#### 3.2.7 Affordability

The cost of services and medicines are a barrier [25, 26, 50, 53, 55, 58]. Regarding indirect costs, only transport costs were reported as an issue [25]. No information was found on how opportunity costs affect the affordability of services.

#### 3.2.8 Ability to Pay

Limited financial resources affect an individual's capacity to pay [25, 26, 34, 55]. No further information was found regarding other potential factors such as assets, social capital or health insurance.

#### 3.2.9 Appropriateness

Researchers have examined the cultural competence of healthcare professionals and its impact on the appropriateness of the healthcare services they provide [25, 26, 28, 47, 50, 51, 57, 58]. Various communication barriers during medical encounters have been studied along with strategies to address them such as language concordant providers, professional interpreters, video remote interpreting, non‐professional interpreters lip‐reading, fingerspelling, writing, texting, visual cues, and video relay interpretation [25, 26, 28, 34, 47, 49, 50, 51, 52, 55, 56, 57, 58]. Each method presents unique benefits and challenges, with access and patience preferences shaped by individual needs, care type, bilingualism and privacy concerns [26, 28, 34, 51, 57]. Some Deaf individuals prefer professional Sign Language interpreter [26, 57], family members (especially for sensitive issues) [28, 51, 57], language‐concordant provider [26, 57], or video remote interpreting [34]. When preferred options are inaccessible, Deaf patients must rely on alternatives such as speech, lip‐reading, fingerspelling or visual cues, which can hinder understanding [26, 50, 57, 58]. Relying on family or friends may create privacy and autonomy concerns, limit understanding of complex medical information, and place an emotional burden on relatives [50].

Service quality is undermined by healthcare professionals' unprofessional behaviour and biases [25, 28, 47, 50, 51, 53, 55, 57, 58]. Communication barriers make it difficult for Deaf patients to build relationships with hearing healthcare staff [26, 34, 52, 54, 58], and are a major reason why patients may not fully understand information provided [51]. Consistency with familiar providers and interpreters is preferred [50, 51]. There is a noted lack of coordination with specialist services who have experience with Deaf patients [50]. Few studies compare treatment for Deaf and hearing individuals [47]. Reviews identified issues including misdiagnosis, incorrect prescriptions [50, 58], insufficient preventative advice [54], violations of health rights [25], and inadequate adaptation of mental health practices [58]. Tools and assessments often lack validation for Deaf populations [26, 50], and health services are frequently ill‐prepared to meet Deaf patients' needs [26, 49].

#### 3.2.10 Ability to Engage

Deaf patients are often excluded from shared decision making due to communication barriers [25, 26, 28, 47, 51, 58, 59], and reliance on family members [25, 53]. They frequently receive inadequate or inaccessible information about procedures, medications, diagnosis and treatments [25, 26, 28, 50, 51, 55, 58], which can discourage them from asking questions and seeking clarification, ultimately affecting their treatment adherence [25, 26, 28, 47, 51]. Their ability to communicate with healthcare professionals depends on their education, reading and speaking skills in a second language, and understanding of medical terminology [25, 26, 28, 49, 57, 59]. The risk of miscommunication is a significant concern among this community [28]. Strong self‐advocacy skills can improve outcomes [28] and community‐based approaches may help develop self‐efficacy [26]. Deaf caregivers also face challenges accessing healthcare services for relatives because of interpreter shortages and inaccessible information [50].

#### 3.2.11 Factor Impacting Access Across Dimensions

One additional factor identified was social inclusion. This factor could not be classified within a specific dimension of access along the utilisation pathway. Instead, it exerted a cross‐sectional influence. This theme was observed in two synthesis [52, 54]. The social inclusion of Deaf individuals informs public policies, which shape the healthcare services available to them [52, 54]. It was further noted that laws and decrees can secure rights for Deaf individuals, leading to improved access [52].

### 3.3 Limitations of Included Studies

The reviews identified limitations in the articles they included. For the quantitative articles, sample sizes were small and/or convenience sampling was used [47, 49]. Hill noted that patient characteristics are highly variable, which affects the ability of interventional studies to effectively control for cofounding variables [47]. Six reviews included a quality assessment [25, 35, 51, 52, 53, 58]. Other authors who didn't conduct a formal quality assessment found that the study design was of variable quality or rigour [34, 56]. Reviews with precise population (e.g., Deaf women, older adults living with dementia) reported limited literature to be inclued [50, 52]. Finally, some researchers noted that the majority of the literature they included came from high‐income countries [26, 57]. Gould and Clark‐Howard noted that the cultural diversity of participants was not always explicitly stated [58].

With regards to the limitations of the reviews, we found that even when the reviews defined their population of interest in their inclusion criteria as Deaf, they included articles that also reported on deaf individuals [25, 26, 34, 47, 49, 54, 55, 57], hard‐of‐hearing individuals [26, 28, 34, 46, 47, 49, 51, 54, 55, 56, 58], people with hearing impairments [25, 51, 53, 56], or people with disabilities [25, 26, 52, 53]. These reviews didn't mention if the data on these groups were excluded from their analysis, except for Rogers et al. [51]. One review noted that the grouping of disabilities in the articles they included did not allow for an analysis of an exclusively Deaf population [53]. Also, none of the reviews included members of the Deaf community in their research teams or mentioned consulting the community. The reviews didn't report whether Deaf researchers and Deaf patients were included in the research teams of their primary articles. Although, one review reported the involvement of a panel of Patient and Public Involvement representatives [50], and one review was co‐conducted by a CODA (child of Deaf adults) [58]. Another notable limitation is that only two reviews considered publications in Sign Language in their inclusion criteria [35, 51]. Of the six systematic reviews, only three included a quality assessment, which may reflect the overall rigour of these reviews [35, 52, 53]. Although it is worth noting that Almeida et al. [52] used the PRISMA reporting guidelines for systematic reviews, which is not a tool that can be used for quality assessment [42].

### 3.4 Research Priorities

The following areas, presented in order of priority, were identified as research priorities for the Deaf community in the province of Québec (Canada): (1) Cost evaluation for the health system and Deaf patients of using (or not using) professional Sign Language interpreters during medical encounters. This cost evaluation should include the costs of treatment delays, unnecessary testing, misdiagnosis, medication errors, complications, adverse events, and consequences for Deaf patients (economic, health and quality of life). (2) Access to emergency medical services. (3) Implementation of a uniform protocol for the use of professional Sign Language interpreters in hospitals. In addition to the protocol, awareness training for healthcare providers on the importance of professional Sign Language interpreters, how to contact interpreting agencies in their area, and how to work alongside with the interpreters. (4) Raising awareness among health science students of Deaf culture, the issues they face in accessing healthcare services, and the needs of the Deaf community. (5) Finding innovative and culturally appropriate solutions to the shortage of qualified professional Sign Language interpreters.

## 4 Discussion

This is the first review of reviews on access to healthcare services for the Deaf, as well as the first to map the studied dimensions of access. The dimensions that have been the most studied are at the two ends of the utilisation pathway: before the perception of need and desire for care (dimensions of approachability and ability to perceive), and after the utilisation of healthcare services (dimension of appropriateness). Our analysis allowed us to further develop the conceptual framework of access to health services by Levesque et al. [5] with the addition of four new factors relevant to the study of access to healthcare services by Deaf populations: communication barriers, cultural competence, technological support, and sociodemographic characteristics. These new factors may be of interest to researchers studying access to healthcare services for other cultural minorities or vulnerable groups. We also identified a cross‐sectional factor that influences multiple dimensions of access: the social inclusion of the Deaf. Social inclusion not only pertains to how policies and accommodations are put in place for the Deaf to have access to healthcare services and facilitate their active participation in society [60]. Another cross‐sectional factor influencing access is the recognition of Deaf language rights. Language rights are part of human rights through the concepts of equality and non‐discrimination in the context of healthcare [61]. In our review, this overarching theme can be identified in eight of the dimensions: approachability, ability to perceive, acceptability, ability to seek, availability and accommodation, affordability, appropriateness, and ability to engage. It was reflected in the lack of health information and services information in Sign Language [28, 47, 50, 51, 56], lack of access to professional interpreters [47, 50, 51, 56], lack of services in Sign Language [25], and lack of accommodation or assistive devices [25]. It was also manifest when Deaf patients were not informed of their right to an interpreter [26, 28, 50]. Health professionals refused to provide professional interpreters [51], used unacceptable forms of communication (lip‐reading, writing) [26, 28, 47, 51, 57], didn't consider confidentiality and privacy issues from a visual language perspective [25, 56], discriminated and neglected Deaf patients [25, 47, 50, 51, 57]. Issues around the recognition of language rights of the Deaf result in Deaf patients not being involved in decision making in their care [25, 47], Deaf patients missing key information to self‐manage their condition [51], and a lower use of preventative services [26, 57]. Multiple Deaf organisations have demanded the recognition of Sign Language as an official language to prevent such a situation [62, 63]. As of today, 81 countries have officially recognised their national Sign Language [62].

Communication barriers and communication methods are the most studied factors compared to other factors, followed by health literacy and the availability of health information. Communication barriers affect patient‐centred care to the point that Deaf patients are discharged without a full understanding of their diagnosis or treatment plan [10, 50, 51, 58, 64]. Studies have raised concerns about consent after Deaf patients reported not receiving information from their healthcare providers or not understanding the information given [12, 13, 50, 64].

With regard to the concentration of the access dimensions studied at the beginning and the end of the utilisation pathway, this could indicate that this is where most of the barriers are experienced. The same could be said of the influence of communication barriers; that most of the challenges and barriers to accessing healthcare services are related to communication barriers. This is supported by the fact that most of the research priorities identified by the Deaf community revolve around communication barriers. However, this interpretation may also be biased by the way in which research on access for Deaf communities is conceptualised and conducted. Firstly, the lack of involvement of Deaf researchers, Deaf patients, and Deaf citizens in the research projects underlines the risk that the available research and possible improvements to healthcare systems are conceptualised from a hearing point of view [34]. Hearing research teams have successfully involved the Deaf community members as co‐researchers [65], and have established a community advisory committee [66]. Researchers have collaborated with the Deaf community and established a permanent community research committee [67]. Deaf communities have also led research initiatives and established research network groups [68, 69, 70, 71]. There is a need for high quality and rigorous research in which Deaf community members are involved in every step of the research projects to ensure that research done on the Deaf community benefits them and is shaped by them.

Only certain thematics of care have been researched, indicating a lack of research in many contexts. For example, no information was identified in this review for patients who are hospitalised where they may face challenges in maintaining access to professional interpreters. There was little or no information for emergency departments or intensive care units, where the urgency of the health status may not allow enough time for healthcare providers to access professional Sign Language interpreters, leaving only the option of using video remote interpreting, delaying care or initiating care without potentially critical information. Communication barriers have had a direct impact on the health of Deaf patients through misdiagnosis, delayed treatment, and unnecessary testing [12, 72]. In turn, critical care healthcare providers may rely on video remote interpreting, but in a study of preferred communication methods in critical and non‐critical care, video remote interpreting was found to be less acceptable to Deaf patients [73]. While access to emergency medical services was identified by the Deaf community as a research priority, we identified no information on emergency medical services. However, this was outside of the scope of our review.

### 4.1 Limitations

The choice of a scoping review of reviews limits the level of analysis of the results presented here, but this approach was considered appropriate for our objectives. Publication and language bias may have influenced our selection process, but we attempted to mediate this effect by including reviews in the American and Quebec Sign Language even if none were identified. This review is also limited by the variable quality of the reviews included. No quality assessment was carried out on the reviews, as our primary aim was to describe the available evidence and this step is not a requirement for a scoping review. We relied solely on author‐led screening during screening of the titles and abstracts. However, to mitigate this potential bias, a pilot round of the title and abstract review was conducted independently by two reviewers on 20 randomly selected articles for standardisation. None of the included syntheses specifically targeted any particular socio‐economic group. Socio‐demographic data regarding the groups involved, as reported in the articles included within these syntheses, were not consistently available; therefore, such information could not be systematically compared across reviews. Finally, as we did not restrict the inclusion of reviews according to a geographical criterion, this limits the generalisability of our findings. We recognise that each Deaf community has a different linguistic and cultural context, but our findings are not intended to be generalised and applied to one Deaf community. We hope that our findings will serve as a starting point for researchers and Deaf community members to engage in discussions and identify research priorities within their community.

## 5 Conclusion

Deaf individuals encounter barriers at every stage of the healthcare utilisation pathway. This review of reviews found that, although all dimensions of access have been examined, numerous influencing factors remain underexplored. Factors such as living environments, transport, and mobility, which may influence the ability to reach services, were not identified in our review. Similarly, aspects affecting the ability to pay—such as assets, social capital, health insurance, and the opportunity costs of accessing services—have not been sufficiently researched. On the demand side, two new themes have emerged: sociodemographic characteristics (relating to the ability to seek care) and technological support (relating to the ability to reach services). Additionally, two cross‐cutting themes—social inclusion and the recognition of language rights—were identified as impacting several dimensions across the utilisation pathway. These findings highlight the necessity for a multi‐level analysis of access for the Deaf community. Researchers should collaborate with local Deaf communities to determine priorities and enhance access to healthcare services for Deaf individuals.

## Author Contributions

**Marie‐Mychèle Pratte:** conceptualisation (lead), project administration (lead), data curation, funding acquisition (lead), investigation (lead), methodology (equal), formal analysis (lead), validation (equal), visualisation, writing – original draft (lead), writing ‐ review and editing (equal). **Magaly Brodeur:** conceptualisation (supporting), funding acquisition (supporting), project administration (supporting), investigation (supporting), methodology (equal), supervision (equal), validation (equal), writing – original draft (supporting), writing ‐ review and editing (equal). **Marie‐Eve Perron:** investigation (supporting), writing ‐ review and editing (equal). **Catherine Hudon:** conceptualisation (supporting), funding acquisition (supporting), project administration (supporting), investigation (supporting), methodology (equal), supervision (equal), validation (equal), writing – original draft (supporting), writing ‐ review and editing (equal).

## Disclosure

The conceptual framework of access to healthcare was published by Levesque et al. [5]. in the International Journal for Equity under a Creative Commons Attribution License (CC BY).

*Use of Generative AI and AI‐Assisted Technologies*: During the writing process, the authors used Copilot (Microsoft) to enhance the quality of the text. After using this tool, the authors have edited and reviewed the content. The authors take full responsibility for the content of this publication.

## Ethics Statement

This scoping review was completed as part of MMP master's degree in health sciences research and was approved by the academic institutional review board at the Université de Sherbrooke (#2024‐4125).

## Consent

The participants of the deliberative workshop signed consent forms available in Quebec Sign Language in video format and written French.

## Conflicts of Interest

The authors declare no conflicts of interest.

## Supporting information

Supporting file 1.

Supporting file 2.

Supporting File 3.

## Data Availability

Data available upon reasonable request, except workshop participants' data for confidentiality purposes (Supporting Information is available as Supporting Information S1–S3: Files S1, S2, S3).
