# Supplementary material for: Access to Healthcare Services for the Deaf: A Scoping Review of Reviews
Source: Health Expect. 2026 Jan 26;29(1):e70554. doi: 10.1111/hex.70554 (PMC12833614; doi:10.1111/hex.70554)
Supplement: Supplementary file 2 — Supporting file 2. [file HEX-29-e70554-s001.docx]

**ACCESS TO HEALTH CARE AND SERVICES FOR THE DEAF:**

**A SCOPING REVIEW OF REVIEWS**

**Supplementary File 2 – Search strings**

***Medline***

| **#** | **Searches** |
| --- | --- |
| 1 | AB ( healthcare OR "health-care" OR "health care" ) OR TI ( healthcare OR "health-care" OR "health care" ) |
| 2 | AB ( ((health OR medical) N3 (service* OR care)) ) OR TI ( ((health OR medical) N3 (service* OR care)) ) |
| 3 | (MH "Health Services+") OR (MH "Delivery of Health Care+") |
| 4 | S1 OR S2 OR S3 |
| 5 | AB ( deaf* OR "D/deaf" ) OR TI ( deaf* OR "D/deaf" ) |
| 6 | AB ( "hard-of-hearing" OR "hard of hearing" OR "hearing-impair*" OR "hearing impair*" OR "hearing-handicap*" OR "hearing handicap*" ) OR TI ( "hard-of-hearing" OR "hard of hearing" OR "hearing-impair*" OR "hearing impair*" OR "hearing-handicap*" OR "hearing handicap*" ) |
| 7 | AB ( HOH OR DHOH OR "D&HH" OR "D&HOH" ) OR TI ( HOH OR DHOH OR "D&HH" OR "D&HOH" ) |
| 8 | AB ( ((hearing OR aural* OR auditor* OR acoustic*) N2 (hard OR impair* OR damage* OR defect* OR difficult* OR loss OR deficien* OR disab* OR handicap* OR disorder* OR mon#aural)) ) OR TI ( ((hearing OR aural* OR auditor* OR acoustic) N2 (hard OR impair* OR damage* OR defect* OR difficult* OR loss OR deficien* OR disab* OR handicap* OR disorder* OR mon#aural)) ) |
| 9 | AB ( presby#c#usi* OR hyp#ac#usi* ) OR TI ( presby#c#usi* OR hyp#ac#usi* ) |
| 10 | AB ( signing OR lipreading OR "lip-reading" OR "lip reading" OR speechreading OR "speech-reading" OR "speech reading" OR gestuno ) OR TI ( signing OR lipreading OR "lip-reading" OR "lip reading" OR speechreading OR "speech-reading" OR "speech reading" OR gestuno ) |
| 11 | AB ( ((sign OR gestural) N3 (language)) ) OR TI ( ((sign OR gestural) N3 (language)) ) |
| 12 | AB ( ((lip* OR speech) N2 (reading OR cued)) ) OR TI ( ((lip* OR speech) N2 (reading OR cued)) ) |
| 13 | AB ( ((visual OR manual) N2 (hearing OR communication)) ) OR TI ( ((visual OR manual) N2 (hearing OR communication)) ) |
| 14 | AB ((total) N2 (communication)) OR TI ((total) N2 (communication)) |
| 15 | AB ( "Conceptually Accurate Signed English" OR pidgin ) OR TI ( "Conceptually Accurate Signed English" OR pidgin ) |
| 16 | (MH "Hearing Disorders+") OR (MH "Persons With Hearing Impairments") OR (MH "Manual Communication+") OR (MH "Lipreading") OR (MH "Communication Methods, Total") OR (MH "Deaf Culture") |
| 17 | S5 OR S6 OR S7 OR S8 OR S9 OR S10 OR S11 OR S12 OR S13 OR S14 OR S15 OR S16 |
| 18 | AB ( review* OR overview* OR synthes* OR scoping ) OR TI ( review* OR overview* OR synthes* OR scoping ) |
| 19 | AB ( "meta-analys*" OR "meta analys*" OR meta#nalys* ) OR TI ( "meta-analys*" OR "meta analys*" OR meta#nalys* ) |
| 20 | AB ( metasynthe* OR "meta-synthe*" OR "meta synthe*" ) OR TI ( metasynthe* OR "meta-synthe*" OR "meta synthe*" ) |
| 21 | AB ( "meta-ethnograph*" OR "meta ethnograph*" ) OR TI ( "meta-ethnograph*" OR "meta ethnograph*" ) |
| 22 | AB ( "meta regression*" OR "meta-regression*" OR metaregression* ) OR TI ( "meta regression*" OR "meta-regression*" OR metaregression* ) |
| 23 | AB ( "meta-aggregation" OR "meta aggregation" ) OR TI ( "meta-aggregation" OR "meta aggregation" ) |
| 24 | AB ( "meta-interpret*" OR "meta interpret*" ) OR TI ( "meta-interpret*" OR "meta interpret*" ) |
| 25 | AB ( "meta-narrative" OR "meta narrative" ) OR TI ( "meta-narrative" OR "meta narrative" ) |
| 26 | AB ( "meta-stud*" OR "meta stud*" ) OR TI ( "meta-stud*" OR "meta stud*" ) |
| 27 | AB ( "state-of-the-art" OR "state of the art" ) OR TI ( "state-of-the-art" OR "state of the art" ) |
| 28 | AB ( "evidence synthe*" OR "evidence report*" ) OR TI ( "evidence synthe*" OR "evidence report*" ) |
| 29 | AB ( "evidence-map*" OR ((evidence) N2 (map*)) ) OR TI ( "evidence-map*" OR ((evidence) N2 (map*)) ) |
| 30 | AB "environmental scan*" OR TI "environmental scan*" |
| 31 | AB ( ((data) N3 (synthes* OR extraction* OR abstraction*)) ) OR TI ( ((data) N3 (synthes* OR extraction* OR abstraction*)) ) |
| 32 | AB ( handsearch* OR "hand search*" ) OR TI ( handsearch* OR "hand search*" ) |
| 33 | AB ( "outcome* research" OR "relative effectiveness" ) OR TI ( "outcome* research" OR "relative effectiveness" ) |
| 34 | AB "horizon scan*" OR TI "horizon scan*" |
| 35 | AB ( ((systematic OR literature OR umbrella OR narrative OR rapid OR structured OR integrative OR comprehensive OR environmental OR map* OR realist* OR research* OR evidence OR collaborative OR concept*) N3 (assess* OR analy*)) ) OR TI ( ((systematic OR literature OR umbrella OR narrative OR rapid OR structured OR integrative OR comprehensive OR environmental OR map* OR realist* OR research* OR evidence OR collaborative OR concept*) N3 (assess* OR analy*)) ) |
| 36 | AB ( ((systematic OR evidence OR synthes*) N3 (research* OR literature OR map*)) ) OR TI ( ((systematic OR evidence OR synthes*) N3 (research* OR literature OR map*)) ) |
| 37 | AB ( ((research*) N3 (integrati* OR overview*)) ) OR TI ( ((research*) N3 (integrati* OR overview*)) ) |
| 38 | AB ( ((pool*) N3 (analy* OR data*)) ) OR TI ( ((pool*) N3 (analy* OR data*)) ) |
| 39 | AB ( ((comparative) N3 (efficacy or effectiveness)) ) OR TI ( ((comparative) N3 (efficacy or effectiveness)) ) |
| 40 | AB ( ((examin* OR summar* OR synthes* OR integrat*) N3 (evidence* OR finding* OR literature*)) ) OR TI ( ((examin* OR summar* OR synthes* OR integrat*) N3 (evidence* OR finding* OR literature*)) ) |
| 41 | AB ( ((comprehensive* OR database* OR electronic* OR "key word*" OR keyword* OR literature* OR method* OR systemati* OR strateg* OR evidence) N3 (search* OR research*)) ) OR TI ( ((comprehensive* OR database* OR electronic* OR "key word*" OR keyword* OR literature* OR method* OR systemati* OR strateg* OR evidence) N3 (search* OR research*)) ) |
| 42 | (MH "Review Literature as Topic+") OR (MH "Meta-Analysis as Topic+") |
| 43 | S18 OR S19 OR S20 OR S21 OR S22 OR S23 OR S24 OR S25 OR S26 OR S27 OR S28 OR S29 OR S30 OR S31 OR S32 OR S33 OR S34 OR S35 OR S36 OR S37 OR S38 OR S39 OR S40 OR S41 OR S42 |
| 44 | S4 AND S17 AND S43 |

***CINAHL***

| **#** | **Searches** |
| --- | --- |
| 1 | AB ( healthcare OR "health-care" OR "health care" ) OR TI ( healthcare OR "health-care" OR "health care" ) |
| 2 | AB ( ((health OR medical) N3 (service* OR care)) ) OR TI ( ((health OR medical) N3 (service* OR care)) ) |
| 3 | (MH "Health Services+") OR (MH "Health Care Delivery+") |
| 4 | S1 OR S2 OR S3 |
| 5 | AB ( deaf* OR "D/deaf" ) OR TI ( deaf* OR "D/deaf" ) |
| 6 | AB ( "hard-of-hearing" OR "hard of hearing" OR "hearing-impair*" OR "hearing impair*" OR "hearing-handicap*" OR "hearing handicap*" ) OR TI ( "hard-of-hearing" OR "hard of hearing" OR "hearing-impair*" OR "hearing impair*" OR "hearing-handicap*" OR "hearing handicap*" ) |
| 7 | AB ( HOH OR DHOH OR "D&HH" OR "D&HOH" ) OR TI ( HOH OR DHOH OR "D&HH" OR "D&HOH" ) |
| 8 | AB ( ((hearing OR aural* OR auditor* OR acoustic*) N2 (hard OR impair* OR damage* OR defect* OR difficult* OR loss OR deficien* OR disab* OR handicap* OR disorder* OR mon#aural)) ) OR TI ( ((hearing OR aural* OR auditor* OR acoustic) N2 (hard OR impair* OR damage* OR defect* OR difficult* OR loss OR deficien* OR disab* OR handicap* OR disorder* OR mon#aural)) ) |
| 9 | AB ( presby#c#usi* OR hyp#ac#usi* ) OR TI ( presby#c#usi* OR hyp#ac#usi* ) |
| 10 | AB ( signing OR lipreading OR "lip-reading" OR "lip reading" OR speechreading OR "speech-reading" OR "speech reading" OR gestuno ) OR TI ( signing OR lipreading OR "lip-reading" OR "lip reading" OR speechreading OR "speech-reading" OR "speech reading" OR gestuno ) |
| 11 | AB ( ((sign OR gestural) N3 (language)) ) OR TI ( ((sign OR gestural) N3 (language)) ) |
| 12 | AB ( ((lip* OR speech) N2 (reading OR cued)) ) OR TI ( ((lip* OR speech) N2 (reading OR cued)) ) |
| 13 | AB ( ((visual OR manual) N2 (hearing OR communication)) ) OR TI ( ((visual OR manual) N2 (hearing OR communication)) ) |
| 14 | AB ((total) N2 (communication)) OR TI ((total) N2 (communication)) |
| 15 | AB ( "Conceptually Accurate Signed English" OR pidgin ) OR TI ( "Conceptually Accurate Signed English" OR pidgin ) |
| 16 | (MH "Hearing Disorders+") OR (MH "Deaf Culture") OR (MH "Sign Language") OR (MH "Lipreading") OR (MH "Communication Methods, Total") |
| 17 | S5 OR S6 OR S7 OR S8 OR S9 OR S10 OR S11 OR S12 OR S13 OR S14 OR S15 OR S16 |
| 18 | AB ( review* OR overview* OR synthes* OR scoping ) OR TI ( review* OR overview* OR synthes* OR scoping ) |
| 19 | AB ( "meta-analys*" OR "meta analys*" OR meta#nalys* ) OR TI ( "meta-analys*" OR "meta analys*" OR meta#nalys* ) |
| 20 | AB ( metasynthe* OR "meta-synthe*" OR "meta synthe*" ) OR TI ( metasynthe* OR "meta-synthe*" OR "meta synthe*" ) |
| 21 | AB ( "meta-ethnograph*" OR "meta ethnograph*" ) OR TI ( "meta-ethnograph*" OR "meta ethnograph*" ) |
| 22 | AB ( "meta regression*" OR "meta-regression*" OR metaregression* ) OR TI ( "meta regression*" OR "meta-regression*" OR metaregression* ) |
| 23 | AB ( "meta-aggregation" OR "meta aggregation" ) OR TI ( "meta-aggregation" OR "meta aggregation" ) |
| 24 | AB ( "meta-interpret*" OR "meta interpret*" ) OR TI ( "meta-interpret*" OR "meta interpret*" ) |
| 25 | AB ( "meta-narrative" OR "meta narrative" ) OR TI ( "meta-narrative" OR "meta narrative" ) |
| 26 | AB ( "meta-stud*" OR "meta stud*" ) OR TI ( "meta-stud*" OR "meta stud*" ) |
| 27 | AB ( "state-of-the-art" OR "state of the art" ) OR TI ( "state-of-the-art" OR "state of the art" ) |
| 28 | AB ( "evidence synthe*" OR "evidence report*" ) OR TI ( "evidence synthe*" OR "evidence report*" ) |
| 29 | AB ( "evidence-map*" OR ((evidence) N2 (map*)) ) OR TI ( "evidence-map*" OR ((evidence) N2 (map*)) ) |
| 30 | AB "environmental scan*" OR TI "environmental scan*" |
| 31 | AB ( ((data) N3 (synthes* OR extraction* OR abstraction*)) ) OR TI ( ((data) N3 (synthes* OR extraction* OR abstraction*)) ) |
| 32 | AB ( handsearch* OR "hand search*" ) OR TI ( handsearch* OR "hand search*" ) |
| 33 | AB ( "outcome* research" OR "relative effectiveness" ) OR TI ( "outcome* research" OR "relative effectiveness" ) |
| 34 | AB "horizon scan*" OR TI "horizon scan*" |
| 35 | AB ( ((systematic OR literature OR umbrella OR narrative OR rapid OR structured OR integrative OR comprehensive OR environmental OR map* OR realist* OR research* OR evidence OR collaborative OR concept*) N3 (assess* OR analy*)) ) OR TI ( ((systematic OR literature OR umbrella OR narrative OR rapid OR structured OR integrative OR comprehensive OR environmental OR map* OR realist* OR research* OR evidence OR collaborative OR concept*) N3 (assess* OR analy*)) ) |
| 36 | AB ( ((systematic OR evidence OR synthes*) N3 (research* OR literature OR map*)) ) OR TI ( ((systematic OR evidence OR synthes*) N3 (research* OR literature OR map*)) ) |
| 37 | AB ( ((research*) N3 (integrati* OR overview*)) ) OR TI ( ((research*) N3 (integrati* OR overview*)) ) |
| 38 | AB ( ((pool*) N3 (analy* OR data*)) ) OR TI ( ((pool*) N3 (analy* OR data*)) ) |
| 39 | AB ( ((comparative) N3 (efficacy or effectiveness)) ) OR TI ( ((comparative) N3 (efficacy or effectiveness)) ) |
| 40 | AB ( ((examin* OR summar* OR synthes* OR integrat*) N3 (evidence* OR finding* OR literature*)) ) OR TI ( ((examin* OR summar* OR synthes* OR integrat*) N3 (evidence* OR finding* OR literature*)) ) |
| 41 | AB ( ((comprehensive* OR database* OR electronic* OR "key word*" OR keyword* OR literature* OR method* OR systemati* OR strateg* OR evidence) N3 (search* OR research*)) ) OR TI ( ((comprehensive* OR database* OR electronic* OR "key word*" OR keyword* OR literature* OR method* OR systemati* OR strateg* OR evidence) N3 (search* OR research*)) ) |
| 42 | (MH "Literature Review+") OR (MH "Meta Analysis") OR (MH "Meta Synthesis") |
| 43 | S18 OR S19 OR S20 OR S21 OR S22 OR S23 OR S24 OR S25 OR S26 OR S27 OR S28 OR S29 OR S30 OR S31 OR S32 OR S33 OR S34 OR S35 OR S36 OR S37 OR S38 OR S39 OR S40 OR S41 OR S42 |
| 44 | S4 AND S17 AND S43 |

***Academic Search Complete***

| # | **Searches** |
| --- | --- |
| 1 | AB ( healthcare OR "health-care" OR "health care" ) OR TI ( healthcare OR "health-care" OR "health care" ) |
| 2 | AB ( ((health OR medical) N3 (service* OR care)) ) OR TI ( ((health OR medical) N3 (service* OR care)) ) |
| 3 | DE "MEDICAL care" OR DE "ACUTE medical care" OR DE "ADVANCE directives (Medical care)" OR DE "ADVERSE health care events" OR DE "CHILD health services" OR DE "CLEFT palate services" OR DE "CLINICAL competence" OR DE "COMMUNITY health services" OR DE "CONSUMER-driven health care" OR DE "CURATIVE medicine" OR DE "DENTAL care" OR DE "DIAGNOSIS" OR DE "DIAGNOSTIC services" OR DE "DIGITAL health" OR DE "DISCRIMINATION in medical care" OR DE "EARLY medical intervention" OR DE "EMERGENCY medical services" OR DE "EMPLOYER health care coalitions" OR DE "FIRST aid in the workplace" OR DE "GENDER specific care" OR DE "HEALTH equity" OR DE "HEALTH facilities" OR DE "HEALTH self-care" OR DE "HEALTH service areas" OR DE "HEALTH services accessibility" OR DE "HETEROSEXISM in medical care" OR DE "HOMOPHOBIA in medical care" OR DE "HOSPITAL care" OR DE "HUMANISTIC medicine" OR DE "INDIVIDUALIZED medicine" OR DE "INTEGRATED health care delivery" OR DE "LONG-term health care" OR DE "MANAGED care programs" OR DE "MEDICAL artifacts" OR DE "MEDICAL care & globalization" OR DE "MEDICAL care use" OR DE "MEDICAL care wait times" OR DE "MEDICAL case management" OR DE "MEDICAL charities" OR DE "MEDICAL compliance" OR DE "MEDICAL screening" OR DE "MEDICAL tourism" OR DE "MEDICALLY underserved areas" OR DE "MEDICALLY underserved persons" OR DE "MEN'S health services" OR DE "MENTAL health services" OR DE "MINIMUM Data Set (Medical Care)" OR DE "MOBILE health" OR DE "NATIONAL health services" OR DE "NURSING services" OR DE "NUTRITION services" OR DE "OCCUPATIONAL health services" OR DE "OCCUPATIONAL therapy services" OR DE "OPTOMETRY" OR DE "OUTPATIENT medical care" OR DE "OVERTREATMENT" OR DE "PARENTAL notification (Medical law)" OR DE "PATIENT acceptance of health care" OR DE "PATIENT care" OR DE "PATIENT-centered care" OR DE "PERIOPERATIVE care" OR DE "PHARMACEUTICAL services" OR DE "PHYSICAL therapy services" OR DE "PHYSICIAN services utilization" OR DE "PRENATAL care" OR DE "PREVENTIVE health services" OR DE "PREVENTIVE medicine" OR DE "PRIMARY care" OR DE "PRIMARY health care" OR DE "REGIONAL medical programs" OR DE "REPRODUCTIVE health services" OR DE "RESOURCE-limited settings" OR DE "RURAL health services" OR DE "SCHOOL health services" OR DE "SECONDARY care (Medicine)" OR DE "SKIN care" OR DE "STANDARDS of care (Gender transition)" OR DE "STUDENT health services" OR DE "SUBACUTE care" OR DE "TERTIARY care" OR DE "TRANSCULTURAL medical care" OR DE "TRANSPHOBIA in medical care" OR DE "TRAUMA-informed care" OR DE "TREATMENT duration" OR DE "UNCOMPENSATED medical care" OR DE "UNDERTREATMENT" OR DE "VETERINARY services" OR DE "VOLUNTEER workers in medical care" OR DE "WOMEN'S health services" OR DE "WOUND care" |
| 4 | DE "ACCESS to primary care" OR DE "MEDICAL care wait times" OR DE "UNIVERSAL healthcare" |
| 5 | DE "SOCIOECONOMIC disparities in health" |
| 6 | S1 OR S2 OR S3 OR S4 OR S5 |
| 7 | AB ( deaf* OR "D/deaf" ) OR TI ( deaf* OR "D/deaf" ) |
| 8 | AB ( "hard-of-hearing" OR "hard of hearing" OR "hearing-impair*" OR "hearing impair*" OR "hearing-handicap*" OR "hearing handicap*" ) OR TI ( "hard-of-hearing" OR "hard of hearing" OR "hearing-impair*" OR "hearing impair*" OR "hearing-handicap*" OR "hearing handicap*" ) |
| 9 | AB ( HOH OR DHOH OR "D&HH" OR "D&HOH" ) OR TI ( HOH OR DHOH OR "D&HH" OR "D&HOH" ) |
| 10 | AB ( ((hearing OR aural* OR auditor* OR acoustic*) N2 (hard OR impair* OR damage* OR defect* OR difficult* OR loss OR deficien* OR disab* OR handicap* OR disorder* OR mon#aural)) ) OR TI ( ((hearing OR aural* OR auditor* OR acoustic) N2 (hard OR impair* OR damage* OR defect* OR difficult* OR loss OR deficien* OR disab* OR handicap* OR disorder* OR mon#aural)) ) |
| 11 | AB ( presby#c#usi* OR hyp#ac#usi* ) OR TI ( presby#c#usi* OR hyp#ac#usi* ) |
| 12 | AB ( signing OR lipreading OR "lip-reading" OR "lip reading" OR speechreading OR "speech-reading" OR "speech reading" OR gestuno ) OR TI ( signing OR lipreading OR "lip-reading" OR "lip reading" OR speechreading OR "speech-reading" OR "speech reading" OR gestuno ) |
| 13 | AB ( ((sign OR gestural) N3 (language)) ) OR TI ( ((sign OR gestural) N3 (language)) ) |
| 14 | AB ( ((lip* OR speech) N2 (reading OR cued)) ) OR TI ( ((lip* OR speech) N2 (reading OR cued)) ) |
| 15 | AB ( ((visual OR manual) N2 (hearing OR communication)) ) OR TI ( ((visual OR manual) N2 (hearing OR communication)) ) |
| 16 | AB ((total) N2 (communication)) OR TI ((total) N2 (communication)) |
| 17 | AB ( "Conceptually Accurate Signed English" OR pidgin ) OR TI ( "Conceptually Accurate Signed English" OR pidgin ) |
| 18 | DE "HEARING disorders" OR DE "ACOUSTIC trauma" OR DE "ALPORT syndrome" OR DE "AUDITORY neuropathy" OR DE "AUDITORY processing disorder" OR DE "CONDUCTIVE hearing loss" OR DE "DEAFNESS" OR DE "HIDDEN hearing loss" OR DE "HIGH-frequency hearing loss" OR DE "HYPERACUSIS" OR DE "KLEIN-Waardenburg syndrome" OR DE "OTOTOXICITY" OR DE "PENDRED syndrome" OR DE "PRESBYCUSIS" OR DE "SENSORINEURAL hearing loss" OR DE "SUPERIOR semicircular canal dehiscence syndrome" OR DE "TINNITUS" |
| 19 | DE "CORTICAL deafness" OR DE "NOISE-induced deafness" OR DE "POSTLINGUAL deafness" |
| 20 | DE "HEARING impaired" OR DE "ACCESSIBLE design for the hearing impaired" OR DE "DEAF people" OR DE "HEARING impaired children" OR DE "LIBRARIES & the hearing impaired" |
| 21 | DE "CHRISTIAN education of the deaf" OR DE "DEAF actors" OR DE "DEAF children" OR DE "DEAF men" OR DE "DEAF parents" OR DE "DEAF poets" OR DE "DEAF teachers" OR DE "DEAF women" OR DE "LIBRARIES & the deaf" OR DE "MUSEUMS & the deaf" OR DE "OLDER deaf people" OR DE "TEACHERS of the deaf" OR DE "TELEVISION & the deaf" |
| 22 | DE "MEANS of communication for deaf people" OR DE "CUED speech" OR DE "FINGER spelling" OR DE "HEARING ear dogs" OR DE "INTERPRETERS for the deaf" OR DE "LIPREADING" OR DE "TELECOMMUNICATIONS devices for the deaf" |
| 23 | DE "SIGN language" OR DE "AMERICAN Sign Language" OR DE "ARGENTINE Sign Language" OR DE "AUSTRALASIAN Signed English" OR DE "AUSTRALIAN Sign Language" OR DE "AUSTRIAN Sign Language" OR DE "BABY signing (Sign language)" OR DE "BAN Khor Sign Language" OR DE "BRAZILIAN Sign Language" OR DE "BRITISH Sign Language" OR DE "CATALAN Sign Language" OR DE "CHILEAN Sign Language" OR DE "CHINESE Sign Language" OR DE "COLOMBIAN Sign Language" OR DE "CZECH Sign Language" OR DE "DANISH Sign Language" OR DE "DUTCH Sign Language" OR DE "ESTONIAN Sign Language" OR DE "FLEMISH Sign Language" OR DE "FRENCH Belgian Sign Language" OR DE "FRENCH Sign Language" OR DE "GERMAN Sign Language" OR DE "GREEK Sign Language" OR DE "HAUSA Sign Language" OR DE "ICELANDIC Sign Language" OR DE "INDONESIAN Sign Language" OR DE "INDOPAKISTAN Sign Language" OR DE "IRISH Sign Language" OR DE "ISRAELI Sign Language" OR DE "ITALIAN Sign Language" OR DE "JAPANESE Sign Language" OR DE "JORDANIAN Sign Language" OR DE "KENYAN Sign Language" OR DE "KOREAN Sign Language" OR DE "MAKATON (Sign language)" OR DE "MEXICAN Sign Language" OR DE "NATIVE American sign language" OR DE "NEW Zealand Sign Language" OR DE "NICARAGUAN Sign Language" OR DE "NORWEGIAN Sign Language" OR DE "PAKISTAN Sign Language" OR DE "PORTUGUESE Sign Language" OR DE "QUEBEC Sign Language" OR DE "SIGNED English" OR DE "SOUTH African Sign Language" OR DE "SPANISH Sign Language" OR DE "SWEDISH Sign Language" OR DE "TAIWAN Sign Language" OR DE "THAI Sign Language" OR DE "VENEZUELAN Sign Language" |
| 24 | DE "DEAF culture" |
| 25 | S7 OR S8 OR S9 OR S10 OR S11 OR S12 OR S13 OR S14 OR S15 OR S16 OR S17 OR S18 OR S19 OR S20 OR S21 OR S22 OR S23 OR S24 |
| 26 | AB ( review* OR overview* OR synthes* OR scoping ) OR TI ( review* OR overview* OR synthes* OR scoping ) |
| 27 | AB ( "meta-analys*" OR "meta analys*" OR meta#nalys* ) OR TI ( "meta-analys*" OR "meta analys*" OR meta#nalys* ) |
| 28 | AB ( metasynthe* OR "meta-synthe*" OR "meta synthe*" ) OR TI ( metasynthe* OR "meta-synthe*" OR "meta synthe*" ) |
| 29 | AB ( "meta-ethnograph*" OR "meta ethnograph*" ) OR TI ( "meta-ethnograph*" OR "meta ethnograph*" ) |
| 30 | AB ( "meta regression*" OR "meta-regression*" OR metaregression* ) OR TI ( "meta regression*" OR "meta-regression*" OR metaregression* ) |
| 31 | AB ( "meta-aggregation" OR "meta aggregation" ) OR TI ( "meta-aggregation" OR "meta aggregation" ) |
| 32 | AB ( "meta-interpret*" OR "meta interpret*" ) OR TI ( "meta-interpret*" OR "meta interpret*" ) |
| 33 | AB ( "meta-narrative" OR "meta narrative" ) OR TI ( "meta-narrative" OR "meta narrative" ) |
| 34 | AB ( "meta-stud*" OR "meta stud*" ) OR TI ( "meta-stud*" OR "meta stud*" ) |
| 35 | AB ( "state-of-the-art" OR "state of the art" ) OR TI ( "state-of-the-art" OR "state of the art" ) |
| 36 | AB ( "evidence synthe*" OR "evidence report*" ) OR TI ( "evidence synthe*" OR "evidence report*" ) |
| 37 | AB ( "evidence-map*" OR ((evidence) N2 (map*)) ) OR TI ( "evidence-map*" OR ((evidence) N2 (map*)) ) |
| 38 | AB "environmental scan*" OR TI "environmental scan*" |
| 39 | AB ( ((data) N3 (synthes* OR extraction* OR abstraction*)) ) OR TI ( ((data) N3 (synthes* OR extraction* OR abstraction*)) ) |
| 40 | AB ( handsearch* OR "hand search*" ) OR TI ( handsearch* OR "hand search*" ) |
| 41 | AB ( "outcome* research" OR "relative effectiveness" ) OR TI ( "outcome* research" OR "relative effectiveness" ) |
| 42 | AB "horizon scan*" OR TI "horizon scan*" |
| 43 | AB ( ((systematic OR literature OR umbrella OR narrative OR rapid OR structured OR integrative OR comprehensive OR environmental OR map* OR realist* OR research* OR evidence OR collaborative OR concept*) N3 (assess* OR analy*)) ) OR TI ( ((systematic OR literature OR umbrella OR narrative OR rapid OR structured OR integrative OR comprehensive OR environmental OR map* OR realist* OR research* OR evidence OR collaborative OR concept*) N3 (assess* OR analy*)) ) |
| 44 | AB ( ((systematic OR evidence OR synthes*) N3 (research* OR literature OR map*)) ) OR TI ( ((systematic OR evidence OR synthes*) N3 (research* OR literature OR map*)) ) |
| 45 | AB ( ((research*) N3 (integrati* OR overview*)) ) OR TI ( ((research*) N3 (integrati* OR overview*)) ) |
| 46 | AB ( ((pool*) N3 (analy* OR data*)) ) OR TI ( ((pool*) N3 (analy* OR data*)) ) |
| 47 | AB ( ((comparative) N3 (efficacy or effectiveness)) ) OR TI ( ((comparative) N3 (efficacy or effectiveness)) ) |
| 48 | AB ( ((examin* OR summar* OR synthes* OR integrat*) N3 (evidence* OR finding* OR literature*)) ) OR TI ( ((examin* OR summar* OR synthes* OR integrat*) N3 (evidence* OR finding* OR literature*)) ) |
| 49 | AB ( ((comprehensive* OR database* OR electronic* OR "key word*" OR keyword* OR literature* OR method* OR systemati* OR strateg* OR evidence) N3 (search* OR research*)) ) OR TI ( ((comprehensive* OR database* OR electronic* OR "key word*" OR keyword* OR literature* OR method* OR systemati* OR strateg* OR evidence) N3 (search* OR research*)) ) |
| 50 | DE "LITERATURE reviews" |
| 51 | DE "SYSTEMATIC reviews" |
| 52 | DE "META-analysis" |
| 53 | DE "META-synthesis" |
| 54 | S26 OR S27 OR S28 OR S29 OR S30 OR S31 OR S32 OR S33 OR S34 OR S35 OR S36 OR S37 OR S38 OR S39 OR S40 OR S41 OR S42 OR S43 OR S44 OR S45 OR S46 OR S47 OR S48 OR S49 OR S50 OR S51 OR S52 OR S53 |
| 55 | S6 AND S25 AND S54 |

***APA PsycInfo***

| **#** | **Searches** |
| --- | --- |
| 1 | AB ( healthcare OR "health-care" OR "health care" ) OR TI ( healthcare OR "health-care" OR "health care" ) |
| 2 | AB ( ((health OR medical) N3 (service* OR care)) ) OR TI ( ((health OR medical) N3 (service* OR care)) ) |
| 3 | DE "Health Care Services" OR DE "Behavioral Health Services" OR DE "Continuum of Care" OR DE "Gender Affirming Care" OR DE "Health Care Delivery" OR DE "Health Screening" OR DE "Hospice" OR DE "Hospital Programs" OR DE "Long Term Care" OR DE "Mental Health Services" OR DE "Palliative Care" OR DE "Patient Centered Care" OR DE "Primary Health Care" OR DE "Reproductive Health Care" OR DE "Social Prescribing" OR DE "Spiritual Care" |
| 4 | DE "Health Care Access" OR DE "Health Care Costs" OR DE "Health Care Reform" OR DE "Health Care Utilization" OR DE "Health Disparities" OR DE "Health Service Needs" OR DE "Managed Care" OR DE "Quality of Care" OR DE "Quality of Services" |
| 5 | DE "Treatment Barriers" |
| 6 | S1 OR S2 OR S3 OR S4 OR S5 |
| 7 | AB ( deaf* OR "D/deaf" ) OR TI ( deaf* OR "D/deaf" ) |
| 8 | AB ( "hard-of-hearing" OR "hard of hearing" OR "hearing-impair*" OR "hearing impair*" OR "hearing-handicap*" OR "hearing handicap*" ) OR TI ( "hard-of-hearing" OR "hard of hearing" OR "hearing-impair*" OR "hearing impair*" OR "hearing-handicap*" OR "hearing handicap*" ) |
| 9 | AB ( HOH OR DHOH OR "D&HH" OR "D&HOH" ) OR TI ( HOH OR DHOH OR "D&HH" OR "D&HOH" ) |
| 10 | AB ( ((hearing OR aural* OR auditor* OR acoustic*) N2 (hard OR impair* OR damage* OR defect* OR difficult* OR loss OR deficien* OR disab* OR handicap* OR disorder* OR mon#aural)) ) OR TI ( ((hearing OR aural* OR auditor* OR acoustic) N2 (hard OR impair* OR damage* OR defect* OR difficult* OR loss OR deficien* OR disab* OR handicap* OR disorder* OR mon#aural)) ) |
| 11 | AB ( presby#c#usi* OR hyp#ac#usi* ) OR TI ( presby#c#usi* OR hyp#ac#usi* ) |
| 12 | AB ( signing OR lipreading OR "lip-reading" OR "lip reading" OR speechreading OR "speech-reading" OR "speech reading" OR gestuno ) OR TI ( signing OR lipreading OR "lip-reading" OR "lip reading" OR speechreading OR "speech-reading" OR "speech reading" OR gestuno ) |
| 13 | AB ( ((sign OR gestural) N3 (language)) ) OR TI ( ((sign OR gestural) N3 (language)) ) |
| 14 | AB ( ((lip* OR speech) N2 (reading OR cued)) ) OR TI ( ((lip* OR speech) N2 (reading OR cued)) ) |
| 15 | AB ( ((visual OR manual) N2 (hearing OR communication)) ) OR TI ( ((visual OR manual) N2 (hearing OR communication)) ) |
| 16 | AB ((total) N2 (communication)) OR TI ((total) N2 (communication)) |
| 17 | AB ( "Conceptually Accurate Signed English" OR pidgin ) OR TI ( "Conceptually Accurate Signed English" OR pidgin ) |
| 18 | DE "Hearing Disorders" OR DE "Hearing Loss" |
| 19 | DE "Deafness" OR DE "Deaf Blind" |
| 20 | DE "Manual Communication" OR DE "Fingerspelling" OR DE "Sign Language" |
| 21 | DE "Lipreading" |
| 22 | S7 OR S8 OR S9 OR S10 OR S11 OR S12 OR S13 OR S14 OR S15 OR S16 OR S17 OR S18 OR S19 OR S20 OR S21 |
| 23 | AB ( review* OR overview* OR synthes* OR scoping ) OR TI ( review* OR overview* OR synthes* OR scoping ) |
| 24 | AB ( "meta-analys*" OR "meta analys*" OR meta#nalys* ) OR TI ( "meta-analys*" OR "meta analys*" OR meta#nalys* ) |
| 25 | AB ( metasynthe* OR "meta-synthe*" OR "meta synthe*" ) OR TI ( metasynthe* OR "meta-synthe*" OR "meta synthe*" ) |
| 26 | AB ( "meta-ethnograph*" OR "meta ethnograph*" ) OR TI ( "meta-ethnograph*" OR "meta ethnograph*" ) |
| 27 | AB ( "meta regression*" OR "meta-regression*" OR metaregression* ) OR TI ( "meta regression*" OR "meta-regression*" OR metaregression* ) |
| 28 | AB ( "meta-aggregation" OR "meta aggregation" ) OR TI ( "meta-aggregation" OR "meta aggregation" ) |
| 29 | AB ( "meta-interpret*" OR "meta interpret*" ) OR TI ( "meta-interpret*" OR "meta interpret*" ) |
| 30 | AB ( "meta-narrative" OR "meta narrative" ) OR TI ( "meta-narrative" OR "meta narrative" ) |
| 31 | AB ( "meta-stud*" OR "meta stud*" ) OR TI ( "meta-stud*" OR "meta stud*" ) |
| 32 | AB ( "state-of-the-art" OR "state of the art" ) OR TI ( "state-of-the-art" OR "state of the art" ) |
| 33 | AB ( "evidence synthe*" OR "evidence report*" ) OR TI ( "evidence synthe*" OR "evidence report*" ) |
| 34 | AB ( "evidence-map*" OR ((evidence) N2 (map*)) ) OR TI ( "evidence-map*" OR ((evidence) N2 (map*)) ) |
| 35 | AB "environmental scan*" OR TI "environmental scan*" |
| 36 | AB ( ((data) N3 (synthes* OR extraction* OR abstraction*)) ) OR TI ( ((data) N3 (synthes* OR extraction* OR abstraction*)) ) |
| 37 | AB ( handsearch* OR "hand search*" ) OR TI ( handsearch* OR "hand search*" ) |
| 38 | AB ( "outcome* research" OR "relative effectiveness" ) OR TI ( "outcome* research" OR "relative effectiveness" ) |
| 39 | AB "horizon scan*" OR TI "horizon scan*" |
| 40 | AB ( ((systematic OR literature OR umbrella OR narrative OR rapid OR structured OR integrative OR comprehensive OR environmental OR map* OR realist* OR research* OR evidence OR collaborative OR concept*) N3 (assess* OR analy*)) ) OR TI ( ((systematic OR literature OR umbrella OR narrative OR rapid OR structured OR integrative OR comprehensive OR environmental OR map* OR realist* OR research* OR evidence OR collaborative OR concept*) N3 (assess* OR analy*)) ) |
| 41 | AB ( ((systematic OR evidence OR synthes*) N3 (research* OR literature OR map*)) ) OR TI ( ((systematic OR evidence OR synthes*) N3 (research* OR literature OR map*)) ) |
| 42 | AB ( ((research*) N3 (integrati* OR overview*)) ) OR TI ( ((research*) N3 (integrati* OR overview*)) ) |
| 43 | AB ( ((pool*) N3 (analy* OR data*)) ) OR TI ( ((pool*) N3 (analy* OR data*)) ) |
| 44 | AB ( ((comparative) N3 (efficacy or effectiveness)) ) OR TI ( ((comparative) N3 (efficacy or effectiveness)) ) |
| 45 | AB ( ((examin* OR summar* OR synthes* OR integrat*) N3 (evidence* OR finding* OR literature*)) ) OR TI ( ((examin* OR summar* OR synthes* OR integrat*) N3 (evidence* OR finding* OR literature*)) ) |
| 46 | AB ( ((comprehensive* OR database* OR electronic* OR "key word*" OR keyword* OR literature* OR method* OR systemati* OR strateg* OR evidence) N3 (search* OR research*)) ) OR TI ( ((comprehensive* OR database* OR electronic* OR "key word*" OR keyword* OR literature* OR method* OR systemati* OR strateg* OR evidence) N3 (search* OR research*)) ) |
| 47 | DE "Literature Review" OR DE "Systematic Review" |
| 48 | DE "Meta Analysis" |
| 49 | S23 OR S24 OR S25 OR S26 OR S27 OR S28 OR S29 OR S30 OR S31 OR S32 OR S33 OR S34 OR S35 OR S36 OR S37 OR S38 OR S39 OR S40 OR S41 OR S42 OR S43 OR S44 OR S45 OR S46 OR S47 OR S48 |
| 50 | S6 AND S22 AND S49 |

***AMED***

| **#** | **Searches** |
| --- | --- |
| 1 | AB ( healthcare OR "health-care" OR "health care" ) OR TI ( healthcare OR "health-care" OR "health care" ) |
| 2 | AB ( ((health OR medical) N3 (service* OR care)) ) OR TI ( ((health OR medical) N3 (service* OR care)) ) |
| 3 | (ZU "health services") or (ZU "health services accessibility") or (ZU "health services needs and demand") or (ZU "delivery of health care") or (ZU "health equity") |
| 4 | S1 OR S2 OR S3 |
| 5 | AB ( deaf* OR "D/deaf" ) OR TI ( deaf* OR "D/deaf" ) |
| 6 | AB ( "hard-of-hearing" OR "hard of hearing" OR "hearing-impair*" OR "hearing impair*" OR "hearing-handicap*" OR "hearing handicap*" ) OR TI ( "hard-of-hearing" OR "hard of hearing" OR "hearing-impair*" OR "hearing impair*" OR "hearing-handicap*" OR "hearing handicap*" ) |
| 7 | AB ( HOH OR DHOH OR "D&HH" OR "D&HOH" ) OR TI ( HOH OR DHOH OR "D&HH" OR "D&HOH" ) |
| 8 | AB ( ((hearing OR aural* OR auditor* OR acoustic*) N2 (hard OR impair* OR damage* OR defect* OR difficult* OR loss OR deficien* OR disab* OR handicap* OR disorder* OR mon#aural)) ) OR TI ( ((hearing OR aural* OR auditor* OR acoustic) N2 (hard OR impair* OR damage* OR defect* OR difficult* OR loss OR deficien* OR disab* OR handicap* OR disorder* OR mon#aural)) ) |
| 9 | AB ( presby#c#usi* OR hyp#ac#usi* ) OR TI ( presby#c#usi* OR hyp#ac#usi* ) |
| 10 | AB ( signing OR lipreading OR "lip-reading" OR "lip reading" OR speechreading OR "speech-reading" OR "speech reading" OR gestuno ) OR TI ( signing OR lipreading OR "lip-reading" OR "lip reading" OR speechreading OR "speech-reading" OR "speech reading" OR gestuno ) |
| 11 | AB ( ((sign OR gestural) N3 (language)) ) OR TI ( ((sign OR gestural) N3 (language)) ) |
| 12 | AB ( ((lip* OR speech) N2 (reading OR cued)) ) OR TI ( ((lip* OR speech) N2 (reading OR cued)) ) |
| 13 | AB ( ((visual OR manual) N2 (hearing OR communication)) ) OR TI ( ((visual OR manual) N2 (hearing OR communication)) ) |
| 14 | AB ((total) N2 (communication)) OR TI ((total) N2 (communication)) |
| 15 | AB ( "Conceptually Accurate Signed English" OR pidgin ) OR TI ( "Conceptually Accurate Signed English" OR pidgin ) |
| 16 | (ZU "deafness") or (ZU "hearing loss") or (ZU "hearing disorders") or (ZU "hearing impaired persons") or (ZU "presbycusis") or (ZU "hearing loss sensorineural") or (ZU "sign language") or (ZU "lipreading") or (ZU "manual communication") |
| 17 | S5 OR S6 OR S7 OR S8 OR S9 OR S10 OR S11 OR S12 OR S13 OR S14 OR S15 OR S16 |
| 18 | AB ( review* OR overview* OR synthes* OR scoping ) OR TI ( review* OR overview* OR synthes* OR scoping ) |
| 19 | AB ( "meta-analys*" OR "meta analys*" OR meta#nalys* ) OR TI ( "meta-analys*" OR "meta analys*" OR meta#nalys* ) |
| 20 | AB ( metasynthe* OR "meta-synthe*" OR "meta synthe*" ) OR TI ( metasynthe* OR "meta-synthe*" OR "meta synthe*" ) |
| 21 | AB ( "meta-ethnograph*" OR "meta ethnograph*" ) OR TI ( "meta-ethnograph*" OR "meta ethnograph*" ) |
| 22 | AB ( "meta regression*" OR "meta-regression*" OR metaregression* ) OR TI ( "meta regression*" OR "meta-regression*" OR metaregression* ) |
| 23 | AB ( "meta-aggregation" OR "meta aggregation" ) OR TI ( "meta-aggregation" OR "meta aggregation" ) |
| 24 | AB ( "meta-interpret*" OR "meta interpret*" ) OR TI ( "meta-interpret*" OR "meta interpret*" ) |
| 25 | AB ( "meta-narrative" OR "meta narrative" ) OR TI ( "meta-narrative" OR "meta narrative" ) |
| 26 | AB ( "meta-stud*" OR "meta stud*" ) OR TI ( "meta-stud*" OR "meta stud*" ) |
| 27 | AB ( "state-of-the-art" OR "state of the art" ) OR TI ( "state-of-the-art" OR "state of the art" ) |
| 28 | AB ( "evidence synthe*" OR "evidence report*" ) OR TI ( "evidence synthe*" OR "evidence report*" ) |
| 29 | AB ( "evidence-map*" OR ((evidence) N2 (map*)) ) OR TI ( "evidence-map*" OR ((evidence) N2 (map*)) ) |
| 30 | AB "environmental scan*" OR TI "environmental scan*" |
| 31 | AB ( ((data) N3 (synthes* OR extraction* OR abstraction*)) ) OR TI ( ((data) N3 (synthes* OR extraction* OR abstraction*)) ) |
| 32 | AB ( handsearch* OR "hand search*" ) OR TI ( handsearch* OR "hand search*" ) |
| 33 | AB ( "outcome* research" OR "relative effectiveness" ) OR TI ( "outcome* research" OR "relative effectiveness" ) |
| 34 | AB "horizon scan*" OR TI "horizon scan*" |
| 35 | AB ( ((systematic OR literature OR umbrella OR narrative OR rapid OR structured OR integrative OR comprehensive OR environmental OR map* OR realist* OR research* OR evidence OR collaborative OR concept*) N3 (assess* OR analy*)) ) OR TI ( ((systematic OR literature OR umbrella OR narrative OR rapid OR structured OR integrative OR comprehensive OR environmental OR map* OR realist* OR research* OR evidence OR collaborative OR concept*) N3 (assess* OR analy*)) ) |
| 36 | AB ( ((systematic OR evidence OR synthes*) N3 (research* OR literature OR map*)) ) OR TI ( ((systematic OR evidence OR synthes*) N3 (research* OR literature OR map*)) ) |
| 37 | AB ( ((research*) N3 (integrati* OR overview*)) ) OR TI ( ((research*) N3 (integrati* OR overview*)) ) |
| 38 | AB ( ((pool*) N3 (analy* OR data*)) ) OR TI ( ((pool*) N3 (analy* OR data*)) ) |
| 39 | AB ( ((comparative) N3 (efficacy or effectiveness)) ) OR TI ( ((comparative) N3 (efficacy or effectiveness)) ) |
| 40 | AB ( ((examin* OR summar* OR synthes* OR integrat*) N3 (evidence* OR finding* OR literature*)) ) OR TI ( ((examin* OR summar* OR synthes* OR integrat*) N3 (evidence* OR finding* OR literature*)) ) |
| 41 | AB ( ((comprehensive* OR database* OR electronic* OR "key word*" OR keyword* OR literature* OR method* OR systemati* OR strateg* OR evidence) N3 (search* OR research*)) ) OR TI ( ((comprehensive* OR database* OR electronic* OR "key word*" OR keyword* OR literature* OR method* OR systemati* OR strateg* OR evidence) N3 (search* OR research*)) ) |
| 42 | (ZU "review literature") or (ZU "review literature as topic") or (ZU "systematic reviews as topic") or (ZU "meta analysis") |
| 43 | S18 OR S19 OR S20 OR S21 OR S22 OR S23 OR S24 OR S25 OR S26 OR S27 OR S28 OR S29 OR S30 OR S31 OR S32 OR S33 OR S34 OR S35 OR S36 OR S37 OR S38 OR S39 OR S40 OR S41 OR S42 |
| 44 | S4 AND S17 AND S43 |

***Healthcare Administration Database***

| **#** | **Searches** |
| --- | --- |
| 1 | healthcare OR "health-care" OR "health care" |
| 2 | ((health OR medical) NEAR/3 (service* OR care)) |
| 3 | MAINSUBJECT.EXACT("Gender-affirming care") OR MAINSUBJECT.EXACT("Health care delivery") OR MAINSUBJECT.EXACT("Health care policy") OR MAINSUBJECT.EXACT("Indigent care") OR MAINSUBJECT.EXACT("Therapy") OR MAINSUBJECT.EXACT("Perioperative care") OR MAINSUBJECT.EXACT("Hospice care") OR MAINSUBJECT.EXACT("Long term health care") OR MAINSUBJECT.EXACT("Nutrition therapy") OR MAINSUBJECT.EXACT("Patient care planning") OR MAINSUBJECT.EXACT("Out of province health care") OR MAINSUBJECT.EXACT("Community health care") OR MAINSUBJECT.EXACT("First aid") OR MAINSUBJECT.EXACT("Preventive medicine") OR MAINSUBJECT.EXACT("Dental care") OR MAINSUBJECT.EXACT("Disease management") OR MAINSUBJECT.EXACT("Emergency medical care") OR MAINSUBJECT.EXACT("Enteral nutrition") OR MAINSUBJECT.EXACT("Prenatal care") OR MAINSUBJECT.EXACT("Telemedicine") OR MAINSUBJECT.EXACT("Health care") OR MAINSUBJECT.EXACT("Health services") OR MAINSUBJECT.EXACT("Medical procedures") OR MAINSUBJECT.EXACT("Medical tourism") OR MAINSUBJECT.EXACT("Wound healing") OR MAINSUBJECT.EXACT("Pain management") OR MAINSUBJECT.EXACT("Respite care") OR MAINSUBJECT.EXACT("Neonatal care") OR MAINSUBJECT.EXACT("Subacute care") OR MAINSUBJECT.EXACT("Symptom management") OR MAINSUBJECT.EXACT("Urban health care") OR MAINSUBJECT.EXACT("Palliative care") OR MAINSUBJECT.EXACT("Rural health care") OR MAINSUBJECT.EXACT("Critical care") OR MAINSUBJECT.EXACT("Nursing care") OR MAINSUBJECT.EXACT("Intensive care") OR MAINSUBJECT.EXACT("Inpatient care") OR MAINSUBJECT.EXACT("Trauma care") OR MAINSUBJECT.EXACT("Health care access") OR MAINSUBJECT.EXACT("Parenteral nutrition") OR MAINSUBJECT.EXACT("Drug therapy") OR MAINSUBJECT.EXACT("Skin care") OR MAINSUBJECT.EXACT("Perinatal care") OR MAINSUBJECT.EXACT("Physical examinations") OR MAINSUBJECT.EXACT("Medical aid") OR MAINSUBJECT.EXACT("Value-based care") OR MAINSUBJECT.EXACT("Medical diagnosis") OR MAINSUBJECT.EXACT("Medical treatment") OR MAINSUBJECT.EXACT("Mental health care") OR MAINSUBJECT.EXACT("Home health care") OR MAINSUBJECT.EXACT("Patient-centered care") OR MAINSUBJECT.EXACT("Private medical care") |
| 4 | MAINSUBJECT.EXACT("Veterans health care") OR MAINSUBJECT.EXACT("Health visiting") OR MAINSUBJECT.EXACT("Military health care") OR MAINSUBJECT.EXACT("Health services utilization") |
| 5 | [S1] OR [S2] OR [S3] OR [S4] |
| 6 | deaf* OR "D/deaf" |
| 7 | "hard-of-hearing" OR "hard of hearing" OR "hearing-impair*" OR "hearing impair*" OR "hearing-handicap*" OR "hearing handicap*" |
| 8 | HOH OR DHOH OR "D&HH" OR "D&HOH" |
| 9 | ((hearing OR aural* OR auditor* OR acoustic*) NEAR/2 (hard OR impair* OR damage* OR defect* OR difficult* OR loss OR deficien* OR disab* OR handicap* OR disorder* OR mon?aural)) |
| 10 | presby?c?usi* OR hyp?ac?usi* |
| 11 | signing OR lipreading OR "lip-reading" OR "lip reading" OR speechreading OR "speech-reading" OR "speech reading" OR gestuno |
| 12 | ((sign OR gestural) NEAR/3 (language)) |
| 13 | ((lip* OR speech) NEAR/2 (reading OR cued)) |
| 14 | ((visual OR manual) NEAR/2 (hearing OR communication)) |
| 15 | ((total) NEAR/2 (communication)) |
| 16 | "Conceptually Accurate Signed English" OR pidgin |
| 17 | MAINSUBJECT.EXACT("Hearing loss") OR MAINSUBJECT.EXACT("Deafness") |
| 18 | MAINSUBJECT.EXACT("Sign language") |
| 19 | MAINSUBJECT.EXACT("Lipreading") |
| 20 | [S6] OR [S7] OR [S8] OR [S9] OR [S10] OR [S11] OR [S12] OR [S13] OR [S14] OR [S15] OR [S16] OR [S17] OR [S18] OR [S19] |
| 21 | review* OR overview* OR synthes* OR scoping |
| 22 | "meta-analys*" OR "meta analys*" OR meta?nalys* |
| 23 | metasynthe* OR "meta-synthe*" OR "meta synthe*" |
| 24 | "meta-ethnograph*" OR "meta ethnograph*" |
| 25 | "meta regression*" OR "meta-regression*" OR metaregression* |
| 26 | "meta-aggregation" OR "meta aggregation" |
| 27 | "meta-interpret*" OR "meta interpret*" |
| 28 | "meta-narrative" OR "meta narrative" |
| 29 | "meta-stud*" OR "meta stud*" |
| 30 | "state-of-the-art" OR "state of the art" |
| 31 | "evidence synthe*" OR "evidence report*" |
| 32 | "evidence-map*" OR ((evidence) NEAR/2 (map*)) |
| 33 | "environmental scan*" |
| 34 | ((data) NEAR/3 (synthes* OR extraction* OR abstraction*)) |
| 35 | handsearch* OR "hand search*" |
| 36 | "outcome* research" OR "relative effectiveness" |
| 37 | "horizon scan*" |
| 38 | ((systematic OR literature OR umbrella OR narrative OR rapid OR structured OR integrative OR comprehensive OR environmental OR map* OR realist* OR research* OR evidence OR collaborative OR concept*) NEAR/3 (assess* OR analy*)) |
| 39 | ((systematic OR evidence OR synthes*) NEAR/3 (research* OR literature OR map*)) |
| 40 | ((research*) NEAR/3 (integrati* OR overview*)) |
| 41 | ((pool*) NEAR/3 (analy* OR data*)) |
| 42 | ((comparative) NEAR/3 (efficacy or effectiveness)) |
| 43 | ((examin* OR summar* OR synthes* OR integrat*) NEAR/3 (evidence* OR finding* OR literature*)) |
| 44 | ((comprehensive* OR database* OR electronic* OR "key word*" OR keyword* OR literature* OR method* OR systemati* OR strateg* OR evidence) NEAR/3 (search* OR research*)) |
| 45 | MAINSUBJECT.EXACT("Literature reviews") OR MAINSUBJECT.EXACT("Systematic review") |
| 46 | MAINSUBJECT.EXACT("Meta-analysis") |
| 47 | MAINSUBJECT.EXACT("Conceptual analysis") |
| 48 | [S21] OR [S22] OR [S23] OR [S24] OR [S25] OR [S26] OR [S27] OR [S28] OR [S29] OR [S30] OR [S31] OR [S32] OR [S33] OR [S34] OR [S35] OR [S36] OR [S37] OR [S38] OR [S39] OR [S40] OR [S41] OR [S42] OR [S43] OR [S44] OR [S45] OR [S46] OR [S47] |
| 49 | [S5] AND [S20] AND [S48] |

***ABI/INFORM Collection***

| **#** | **Searches** |
| --- | --- |
| 1 | healthcare OR "health-care" OR "health care" |
| 2 | ((health OR medical) NEAR/3 (service* OR care)) |
| 3 | MAINSUBJECT.EXACT("Gender-affirming care") OR MAINSUBJECT.EXACT("Health care delivery") OR MAINSUBJECT.EXACT("Health care policy") OR MAINSUBJECT.EXACT("Indigent care") OR MAINSUBJECT.EXACT("Therapy") OR MAINSUBJECT.EXACT("Perioperative care") OR MAINSUBJECT.EXACT("Hospice care") OR MAINSUBJECT.EXACT("Long term health care") OR MAINSUBJECT.EXACT("Nutrition therapy") OR MAINSUBJECT.EXACT("Patient care planning") OR MAINSUBJECT.EXACT("Out of province health care") OR MAINSUBJECT.EXACT("Community health care") OR MAINSUBJECT.EXACT("First aid") OR MAINSUBJECT.EXACT("Preventive medicine") OR MAINSUBJECT.EXACT("Dental care") OR MAINSUBJECT.EXACT("Disease management") OR MAINSUBJECT.EXACT("Emergency medical care") OR MAINSUBJECT.EXACT("Enteral nutrition") OR MAINSUBJECT.EXACT("Prenatal care") OR MAINSUBJECT.EXACT("Telemedicine") OR MAINSUBJECT.EXACT("Health care") OR MAINSUBJECT.EXACT("Health services") OR MAINSUBJECT.EXACT("Medical procedures") OR MAINSUBJECT.EXACT("Medical tourism") OR MAINSUBJECT.EXACT("Wound healing") OR MAINSUBJECT.EXACT("Pain management") OR MAINSUBJECT.EXACT("Respite care") OR MAINSUBJECT.EXACT("Neonatal care") OR MAINSUBJECT.EXACT("Subacute care") OR MAINSUBJECT.EXACT("Symptom management") OR MAINSUBJECT.EXACT("Urban health care") OR MAINSUBJECT.EXACT("Palliative care") OR MAINSUBJECT.EXACT("Rural health care") OR MAINSUBJECT.EXACT("Critical care") OR MAINSUBJECT.EXACT("Nursing care") OR MAINSUBJECT.EXACT("Intensive care") OR MAINSUBJECT.EXACT("Inpatient care") OR MAINSUBJECT.EXACT("Trauma care") OR MAINSUBJECT.EXACT("Health care access") OR MAINSUBJECT.EXACT("Parenteral nutrition") OR MAINSUBJECT.EXACT("Drug therapy") OR MAINSUBJECT.EXACT("Perinatal care") OR MAINSUBJECT.EXACT("Skin care") OR MAINSUBJECT.EXACT("Physical examinations") OR MAINSUBJECT.EXACT("Medical aid") OR MAINSUBJECT.EXACT("Value-based care") OR MAINSUBJECT.EXACT("Medical diagnosis") OR MAINSUBJECT.EXACT("Medical treatment") OR MAINSUBJECT.EXACT("Mental health care") OR MAINSUBJECT.EXACT("Home health care") OR MAINSUBJECT.EXACT("Patient-centered care") OR MAINSUBJECT.EXACT("Private medical care") |
| 4 | MAINSUBJECT.EXACT("Veterans health care") OR MAINSUBJECT.EXACT("Health visiting") OR MAINSUBJECT.EXACT("Military health care") OR MAINSUBJECT.EXACT("Health services utilization") |
| 5 | [S1] OR [S2] OR [S3] OR [S4] |
| 6 | deaf* OR "D/deaf" |
| 7 | "hard-of-hearing" OR "hard of hearing" OR "hearing-impair*" OR "hearing impair*" OR "hearing-handicap*" OR "hearing handicap*" |
| 8 | HOH OR DHOH OR "D&HH" OR "D&HOH" |
| 9 | ((hearing OR aural* OR auditor* OR acoustic*) NEAR/2 (hard OR impair* OR damage* OR defect* OR difficult* OR loss OR deficien* OR disab* OR handicap* OR disorder* OR mon?aural)) |
| 10 | presby?c?usi* OR hyp?ac?usi* |
| 11 | signing OR lipreading OR "lip-reading" OR "lip reading" OR speechreading OR "speech-reading" OR "speech reading" OR gestuno |
| 12 | ((sign OR gestural) NEAR/3 (language)) |
| 13 | ((lip* OR speech) NEAR/2 (reading OR cued)) |
| 14 | ((visual OR manual) NEAR/2 (hearing OR communication)) |
| 15 | ((total) NEAR/2 (communication)) |
| 16 | "Conceptually Accurate Signed English" OR pidgin |
| 17 | MAINSUBJECT.EXACT("Hearing loss") OR MAINSUBJECT.EXACT("Deafness") |
| 18 | MAINSUBJECT.EXACT("Sign language") |
| 19 | MAINSUBJECT.EXACT("Lipreading") |
| 20 | [S6] OR [S7] OR [S8] OR [S9] OR [S10] OR [S11] OR [S12] OR [S13] OR [S14] OR [S15] OR [S16] OR [S17] OR [S18] OR [S19] |
| 21 | review* OR overview* OR synthes* OR scoping |
| 22 | "meta-analys*" OR "meta analys*" OR meta?nalys* |
| 23 | metasynthe* OR "meta-synthe*" OR "meta synthe*" |
| 24 | "meta-ethnograph*" OR "meta ethnograph*" |
| 25 | "meta regression*" OR "meta-regression*" OR metaregression* |
| 26 | "meta-aggregation" OR "meta aggregation" |
| 27 | "meta-interpret*" OR "meta interpret*" |
| 28 | "meta-narrative" OR "meta narrative" |
| 29 | "meta-stud*" OR "meta stud*" |
| 30 | "state-of-the-art" OR "state of the art" |
| 31 | "evidence synthe*" OR "evidence report*" |
| 32 | "evidence-map*" OR ((evidence) NEAR/2 (map*)) |
| 33 | "environmental scan*" |
| 34 | ((data) NEAR/3 (synthes* OR extraction* OR abstraction*)) |
| 35 | handsearch* OR "hand search*" |
| 36 | "outcome* research" OR "relative effectiveness" |
| 37 | "horizon scan*" |
| 38 | ((systematic OR literature OR umbrella OR narrative OR rapid OR structured OR integrative OR comprehensive OR environmental OR map* OR realist* OR research* OR evidence OR collaborative OR concept*) NEAR/3 (assess* OR analy*)) |
| 39 | ((systematic OR evidence OR synthes*) NEAR/3 (research* OR literature OR map*)) |
| 40 | ((research*) NEAR/3 (integrati* OR overview*)) |
| 41 | ((pool*) NEAR/3 (analy* OR data*)) |
| 42 | ((comparative) NEAR/3 (efficacy or effectiveness)) |
| 43 | ((examin* OR summar* OR synthes* OR integrat*) NEAR/3 (evidence* OR finding* OR literature*)) |
| 44 | ((comprehensive* OR database* OR electronic* OR "key word*" OR keyword* OR literature* OR method* OR systemati* OR strateg* OR evidence) NEAR/3 (search* OR research*)) |
| 45 | MAINSUBJECT.EXACT("Literature reviews") OR MAINSUBJECT.EXACT("Systematic review") |
| 46 | MAINSUBJECT.EXACT("Meta-analysis") |
| 47 | MAINSUBJECT.EXACT("Conceptual analysis") |
| 48 | [S21] OR [S22] OR [S23] OR [S24] OR [S25] OR [S26] OR [S27] OR [S28] OR [S29] OR [S30] OR [S31] OR [S32] OR [S33] OR [S34] OR [S35] OR [S36] OR [S37] OR [S38] OR [S39] OR [S40] OR [S41] OR [S42] OR [S43] OR [S44] OR [S45] OR [S46] OR [S47] |
| 49 | [S5] AND [S20] AND [S48] |

***Cochrane Database of Systematic Reviews***

| **#** | **Searches** |
| --- | --- |
| 1 | healthcare OR "health-care" OR "health care" |
| 2 | ((health OR medical) NEAR/3 (service* OR care)) |
| 3 | (MH "Health Services+") |
| 4 | (MH "Delivery of Health Care+") |
| 5 | #1 OR #2 OR #3 OR #4 |
| 6 | deaf* OR "D/deaf" |
| 7 | "hard-of-hearing" OR "hard of hearing" OR hearing-impair* OR hearing impair* OR hearing-handicap* OR hearing handicap* |
| 8 | HOH OR DHOH OR "D&HH" OR "D&HOH" |
| 9 | ((hearing OR aural* OR auditor* OR acoustic*) NEAR/2 (hard OR impair* OR damage* OR defect* OR difficult* OR loss OR deficien* OR disab* OR handicap* OR disorder* OR mon?aural)) |
| 10 | presby?c?usi* OR hyp?ac?usi* |
| 11 | signing OR lipreading OR "lip-reading" OR "lip reading" OR speechreading OR "speech-reading" OR "speech reading" OR gestuno |
| 12 | ((sign OR gestural) NEAR/3 (language)) |
| 13 | ((lip* OR speech) NEAR/2 (reading OR cued)) |
| 14 | ((visual OR manual) NEAR/2 (hearing OR communication)) |
| 15 | ((total) NEAR/2 (communication)) |
| 16 | "Conceptually Accurate Signed English" OR pidgin |
| 17 | (MH "Hearing Disorders+") |
| 18 | (MH "Persons With Hearing Impairments") |
| 19 | (MH "Manual Communication+") |
| 20 | (MH "Lipreading") |
| 21 | (MH "Communication Methods, Total") |
| 22 | (MH "Deaf Culture") |
| 23 | #6 OR #7 OR #8 OR #9 OR #10 OR #11 OR #12 OR #13 OR #14 OR #15 OR #16 OR #17 OR #18 OR #19 OR #20 OR #21 OR #22 |
| 24 | #5 AND #23 |

***Epistemonikos***

| **#** | **Searches** |
| --- | --- |
| 1 | healthcare OR "health-care" OR "health care" |
| 2 | health service* OR health care OR medical service* OR medical care |
| 3 | S1 OR S2 |
| 4 | deaf* OR "D/deaf" |
| 5 | "hard-of-hearing" OR "hard of hearing" OR "hearing-impair*" OR "hearing impair*" OR "hearing-handicap*" OR "hearing handicap*" |
| 6 | HOH OR DHOH OR "D&HH" OR "D&HOH" |
| 7 | hearing damage* OR hearing defect* OR hearing difficult* OR hearing loss OR hearing deficien* OR hearing disab* OR hearing disorder* OR monoaural hearing OR monaural hearing |
| 8 | aural* impair* OR aural* impair* OR aural* damage* OR aural* defect* OR aural* difficult* OR aural* loss OR aural* deficien* OR aural* disab* OR aural* handicap* OR aural* disorder* |
| 9 | auditor* impair* OR auditor* damage* OR auditor* defect* OR auditor* difficult* OR auditor* loss OR auditor* deficien* OR auditor* disab* OR auditor* handicap* OR auditor* disorder* OR auditor* monoaural OR auditor* monaural |
| 10 | acoustic* damage* OR acoustic* defect* OR acoustic* difficult* OR acoustic* loss OR acoustic* deficien* OR acoustic* disab* OR acoustic* handicap* OR acoustic* disorder* OR acoustic* monoaural OR acoustic*monaural |
| 11 | presbyacusi* OR presbycusi* OR presbyacousi* OR hypoacousi* OR hypoacusi* OR hypacusi* |
| 12 | signing OR lipreading OR "lip-reading" OR "lip reading" OR speechreading OR "speech-reading" OR "speech reading" OR gestuno |
| 13 | sign language OR gestural language |
| 14 | lip* reading OR speech reading OR cued speech |
| 15 | visual hearing OR visual communication OR manual hearing OR manual communication |
| 16 | total communication |
| 17 | "Conceptually Accurate Signed English" OR pidgin |
| 18 | S4 OR S5 OR S6 OR S7 OR S8 OR S9 OR S10 OR S11 OR S12 OR S13 OR S14 OR S15 OR S16 OR S17 |
| 19 | S3 AND S18 |
| 20 | S19 + filter Broad synthesis |
| 21 | S19 + filter Systematic Review |
| 22 | S19 + filter Structured summary of synthesis |

***Scopus***

| **#** | **Searches** |
| --- | --- |
| 1 | healthcare OR "health-care" OR "health care" |
| 2 | ((health OR medical) W/3 (service* OR care)) |
| 3 | S1 OR S2 |
| 4 | deaf* OR "D/deaf" |
| 5 | "hard-of-hearing" OR "hard of hearing" OR "hearing-impair*" OR "hearing impair*" OR "hearing-handicap*" OR "hearing handicap*" |
| 6 | HOH OR DHOH OR "D&HH" OR "D&HOH" |
| 7 | ((hearing OR aural* OR auditor* OR acoustic*) W/2 (hard OR impair* OR damage* OR defect* OR difficult* OR loss OR deficien* OR disab* OR handicap* OR disorder* OR mon?aural)) |
| 8 | presbyacusi* OR presbycusi* OR presbyacousi* OR hypoacousi* OR hypoacusi* OR hypacusi* |
| 9 | signing OR lipreading OR "lip-reading" OR "lip reading" OR speechreading OR "speech-reading" OR "speech reading" OR gestuno |
| 10 | ((sign OR gestural) W/3 (language)) |
| 11 | ((lip* OR speech) W/2 (reading OR cued)) |
| 12 | ((visual OR manual) W/2 (hearing OR communication)) |
| 13 | ((total) W/2 (communication)) |
| 14 | "Conceptually Accurate Signed English" OR pidgin |
| 15 | S4 OR S5 OR S6 OR S7 OR S8 OR S9 OR S10 OR S11 OR S12 OR S13 OR S14 |
| 16 | review* OR overview* OR synthes* OR scoping |
| 17 | "meta-analys*" OR "meta analys*" OR metanalys* OR metaanalys* |
| 18 | metasynthe* OR "meta-synthe*" OR "meta synthe*" |
| 19 | "meta-ethnograph*" OR "meta ethnograph*" |
| 20 | "meta regression*" OR "meta-regression*" OR metaregression* |
| 21 | "meta-aggregation" OR "meta aggregation" |
| 22 | meta-interpret* OR meta interpret* |
| 23 | "meta-narrative" OR "meta narrative" |
| 24 | meta-stud* OR meta stud* |
| 25 | "state-of-the-art" OR "state of the art" |
| 26 | evidence synthe* OR evidence report* |
| 27 | evidence-map* OR ((evidence) W/2 (map*)) |
| 28 | environmental scan* |
| 29 | ((data) W/3 (synthes* OR extraction* OR abstraction*)) |
| 30 | handsearch* OR hand search* |
| 31 | outcome* research OR "relative effectiveness" |
| 32 | horizon scan* |
| 33 | (systematic OR literature OR umbrella OR narrative OR rapid OR structured OR integrative OR comprehensive OR environmental OR map* OR realist* OR research* OR evidence OR collaborative OR concept*) W/3 (assess* OR analy*) |
| 34 | (systematic OR evidence OR synthes*) W/3 (research* OR literature OR map*) |
| 35 | (research*) W/3 (integrati* OR overview*) |
| 36 | (pool*) W/3 (analy* OR data*) |
| 37 | (comparative) W/3 (efficacy or effectiveness) |
| 38 | (examin* OR summar* OR synthes* OR integrat*) W/3 (evidence* OR finding* OR literature*) |
| 39 | (comprehensive* OR database* OR electronic* OR "key word*" OR keyword* OR literature* OR method* OR systemati* OR strateg* OR evidence) W/3 (search* OR research*) |
| 40 | S16 OR S17 OR S18 OR S19 OR S20 OR S21 OR S22 OR S23 OR S24 OR S25 OR S26 OR S27 OR S28 OR S29 OR S30 OR S31 OR S32 OR S33 OR S34 OR S35 OR S36 OR S37 OR S38 OR S39 OR S40 OR S41 OR S42 |
| 41 | S3 AND S15 AND S40 |

***Google Scholar***

| **#** | **Search** |
| --- | --- |
| 1 | deaf AND healthcare AND review |
