# Supplementary material for: Access to Healthcare Services for the Deaf: A Scoping Review of Reviews
Source: Health Expect. 2026 Jan 26;29(1):e70554. doi: 10.1111/hex.70554 (PMC12833614; doi:10.1111/hex.70554)
Supplement: Supplementary file 3 — Supporting File 3. [file HEX-29-e70554-s003.docx]

**ACCESS TO HEALTH CARE AND SERVICES FOR THE DEAF:**

**A SCOPING REVIEW OF REVIEWS**

**Supplementary File 3 – Extraction Form**

**General characteristics**

| Title |  | |
| --- | --- | --- |
| Publication year |  | |
| Country of origin  *(from first author's affiliation)* |  | |
| Language | ( ) English  ( ) French | ( ) ASL  ( ) LSQ |
| Funding source |  | |
| Potential source(s) of conflict of interest |  | |

**Methodology**

|  | *As written in the text* |
| --- | --- |
| Research question(s) |  |
| Research objective(s) |  |
| Type of review | ( ) Integrative review  ( ) Meta-analysis  ( ) Meta-synthesis  ( ) Narrative review  ( ) Rapid review  ( ) Realist review  ( ) Report  ( ) Scoping review  ( ) Systematic review  Other: |
| Type of data analysis |  |
| Inclusion criteria |  |
| Exclusion criteria |  |
| Limits  *(e.g., date range, language, filters)* |  |
| Databases and sources searched |  |
| Was there a quality assessment |  |
| Type of quality assessment |  |

**Population**

|  | *As written in the text* |
| --- | --- |
| Definition of the population *(if present)* |  |
| Notes  *(e.g., specific subgroups)* |  |

**Phenomena of interest**

|  | *As written in the text* | |
| --- | --- | --- |
| Is a definition of access offered? | ( ) Yes | ( ) No |
| Definition of access |  | |
| Dimensions studied | ( ) Access  ( ) Accessibility  ( ) Ability  ( ) Approachability  ( ) Ability to perceive  ( ) Perception of needs and desire for care  ( ) Acceptability  ( ) Ability to seek  ( ) Health care seeking  ( ) Availability and accommodation  ( ) Ability to reach  ( ) Health care reaching  ( ) Affordability  ( ) Ability to pay  ( ) Health care utilization  ( ) Appropriateness  ( ) Ability to engage  ( ) Health care consequences | |
| Specific factors of dimensions  *(Dimension – factor)* |  | |

**Context**

|  | *As written in the text* |
| --- | --- |
| Context of health services | ( ) Health services in general  ( ) Care continuum  ( ) Primary care (or primary health care)  ( ) Secondary care  ( ) Tertiary care  ( ) Not specified  ( ) Other : |
| Notes *(e.g., specialty, department, geographic region, type of intervention)* |  |

**Results**

|  | *As written in the text* |
| --- | --- |
| Number of articles |  |
| Date range |  |
| Countries covered |  |
| Types of studies included |  |
| Themes |  |
| Dimensions studied |  |
| Key recommendations or conclusions |  |
| Strength of review |  |
| Limits of review |  |
| Notes |  |
